# Geriatric Syndromes and Mortality Among Hospitalized Older Adults

**DOI:** 10.1001/jamanetworkopen.2025.55740

**Published:** 2026-01-27

**Authors:** Thiago J. Avelino-Silva, Maria Fernanda B. Roma, Adriana F. Dutra, Alexandra Malheiro, Ana Cristina C. Speranza, Arlety M. C. Casale, Beatriz N. A. Lopes, Clineu M. Almada-Filho, Danilsa V. de Sousa, Eduardo Marques da Silva, Fabiola Sepulveda, Flavia Barreto Garcez, Gabriel T. Constantino, Gabriela S. Keller, Ianna L. S. Braga, Jonas Gordilho Souza, Juliana J. M. Teixeira, Karoline Rodrigues da Silva Martins, Laiane Moraes Dias, Lara M. Q. Araujo, Luana A. C. Macedo, Lucas G. de Andrade, Lucas K. P. Prado, Luis Carlos Venegas-Sanabria, Marco P. D. Freitas, Marcos D. C. Saraiva, Maria Aparecida C. Bicalho, Maria Carolyna F. B. Arbex, Maria E. Pires, Maria M. V. Guedes, Marina M. G. Borges, Milton L. Gorzoni, Mirella R. Bezerra, Natalia I. B. Garção, Natascha G. F. Palmeira, Nereida K. C. Lima, Oberdã G. Moreira-Filho, Paulo José F. Villas Boas, Perola Q. de Almeida, Renata M. Dip, Renato G. Bandeira de Mello, Samir A. Aruachan, Theodora Karnakis, Vitor L. Pintarelli, Welma W. C. C. Amorim, Yngrid Dieguez Ferreira, Kenneth E. Covinsky, Eduardo Ferriolli, Sei J. Lee, Alexander K. Smith, Claudia K. Suemoto, Marlon J. R. Aliberti

**Affiliations:** 1 Laboratorio de Investigacao Medica em Envelhecimento (LIM-66), Serviço de Geriatria, Hospital das Clínicas da Faculdade de Medicina, Universidade de São Paulo, São Paulo, São Paulo, Brazil; 2 Division of Geriatrics, University of California, San Francisco; 3 Hospital das Clínicas da Unicamp, Campinas, São Paulo, Brazil; 4 Hospital do Coração, São Paulo, São Paulo, Brazil; 5 Hospital Lusíadas Porto, Porto, Portugal; 6 Hospital Universitário Pedro Ernesto, Rio de Janeiro, Rio de Janeiro, Brazil; 7 Hospital Universitário da Universidade Federal de São Carlos, São Carlos, São Paulo, Brazil; 8 Hospital do Coração de Natal, Natal, Rio Grande do Norte, Brazil; 9 Hospital São Paulo, Universidade Federal de São Paulo, São Paulo, São Paulo, Brazil; 10 Clínica Girassol, Luanda, Angola; 11 Hospital Emílio Carlos, Centro Universitário Padre Albino, Catanduva, São Paulo, Brazil; 12 Hospital Regional de Talca Dr César Garavagno Burotto, Talca, Chile; 13 Hospital Universitário da Universidade Federal de Sergipe, Aracaju, Sergipe, Brazil; 14 A. C. Camargo Cancer Center, São Paulo, São Paulo, Brazil; 15 Hospital São José, Criciúma, Santa Catarina, Brazil; 16 Hospital Geral Dr César Cals de Oliveira, Fortaleza, Ceará, Brazil; 17 Hospital Universitário Professor Edgard Santos, Salvador, Bahia, Brazil; 18 Hospital de Urgências de Goiás, Goiânia, Goiás, Brazil; 19 Hospital Universitário Getúlio Vargas, Manaus, Amazonas, Brazil; 20 Hospital Regional Jean Bitar, Belém, Pará, Brazil; 21 Hospital Universitário Onofre Lopes, Natal, Rio Grande do Norte, Brazil; 22 Real Hospital Português de Beneficência, Recife, Pernambuco, Brazil; 23 Hospital Santo Antônio–Obras Sociais Irmã Dulce, Salvador, Bahia, Brazil; 24 Hospital Universitario Mayor Méderi, Bogotá, Colombia; 25 Hospital Universitário de Brasília, Brasília, Distrito Federal, Brazil; 26 Hospital Central da Irmandade da Santa Casa de Misericórdia de São Paulo, São Paulo, São Paulo, Brazil; 27 Hospital das Clínicas da Universidade Federal de Minas Gerais, Belo Horizonte, Minas Gerais, Brazil; 28 Santa Casa de Araraquara, Araraquara, São Paulo, Brazil; 29 Hospital Sírio Libanês, São Paulo, São Paulo, Brazil; 30 Instituto do Câncer do Estado de São Paulo, São Paulo, São Paulo, Brazil; 31 Hospital das Clínicas da Universidade Federal de Pernambuco, Recife, Pernambuco, Brazil; 32 Hospital Universitário João de Barros Barreto, Belém, Pará, Brazil; 33 Instituto de Medicina Integral Prof Fernando Figueira, Recife, Pernambuco, Brazil; 34 Beneficência Portuguesa de São Paulo, São Paulo, São Paulo, Brazil; 35 Hospital Israelita Albert Einstein, São Paulo, São Paulo, Brazil; 36 Hospital das Clínicas da Faculdade de Medicina de Ribeirão Preto, Ribeirão Preto, São Paulo, Brazil; 37 Hospital São Camilo, São Paulo, São Paulo, Brazil; 38 Hospital das Clínicas da Faculdade de Medicina de Botucatu, Botucatu, São Paulo, Brazil; 39 Hospital Universitário da Universidade Estadual de Londrina, Londrina, Paraná, Brazil; 40 Hospital de Clínicas de Porto Alegre, Porto Alegre, Rio Grande do Sul, Brazil; 41 Hospital Universitario San Ignacio, Bogotá, Colombia; 42 Hospital Nossa Senhora das Graças, Curitiba, Paraná, Brazil; 43 Hospital Geral de Vitória da Conquista, Vitória da Conquista, Bahia, Brazil; 44 Hospital Geriátrico e de Convalescentes Dom Pedro II, São Paulo, São Paulo, Brazil; 45 Center on Aging and Health, Johns Hopkins Medical Institutions, Baltimore, Maryland

## Abstract

**Importance:**

Geriatric syndromes are common in hospitalized older adults and complicate acute care; however, their overall prevalence and cumulative burden remain poorly understood, especially in resource-limited settings.

**Objectives:**

To measure the prevalence of geriatric syndromes upon hospital admission and examine the independent association between the number of geriatric syndromes and 90-day mortality.

**Design, Setting, and Participants:**

This cohort study used data from the Creating a Hospital Assessment Network in Geriatrics (CHANGE) study, a multicenter, prospective cohort of 43 hospitals, including 38 in Brazil, 1 in Angola, 1 in Chile, 2 in Colombia, and 1 in Portugal. Consecutive patients aged 65 years or older admitted under geriatric teams between June 1, 2022, and December 31, 2023, were enrolled within 48 hours; patients with terminally illness were excluded. Data were analyzed from February 1 to November 23, 2025.

**Exposure:**

A standardized comprehensive geriatric assessment captured 14 geriatric syndromes: loneliness, dementia, depressive symptoms, sensory impairment, disability, immobility, incontinence, falls, frailty, malnutrition, pressure ulcers, polypharmacy, potentially inappropriate medications, and delirium. The exposure of interest was the within-patient count of syndromes.

**Main Outcomes and Measures:**

The primary outcome was 90-day all-cause mortality, ascertained by masked telephone follow-up with verification in medical records or public registries. Prespecified mixed-effects Cox proportional hazards regression were performed.

**Results:**

The study included 2556 participants (mean [SD] age, 79 [9] years, 1437 female [56.2%]). The median number of geriatric syndromes was 5 (IQR, 3-8). The highest prevalence rates for syndromes were 70.8% (95% CI, 69.1%-72.6%) for disability, 61.7% (95% CI, 59.8%-63.6%) for polypharmacy, 58.2% (95% CI, 56.3%-60.1%) for frailty, and 54.7% (95% CI, 52.8%-56.7%) for sensory impairment. Across categories, the mortality rate rose from 8.4% (95% CI, 6.2%-11.4%) for 0 to 2 syndromes to 12.7% (95% CI, 10.1%-15.7%) for 3 to 4 syndromes, 25.4% (95% CI, 22.2%-29.1%) for 5 to 6 syndromes, 30.4% (95% CI, 26.7%-34.5%) for 7 to 8 syndromes, 39.5% (95% CI, 34.4%-44.8%) for 9 to 10 syndromes, and 47.0% (95% CI, 36.4%-57.9%) for 11 or more syndromes. After adjusting for confounders, each additional geriatric syndrome was associated with an increased risk of mortality (hazard ratio, 1.22 [95% CI, 1.15-1.30), which became increasingly pronounced in older age groups.

**Conclusions and Relevance:**

This cohort study found that hospitalized older adults had a median of 5 geriatric syndromes, which were independently and incrementally associated with 90-day mortality. Multidomain assessments should be integrated into standard hospital care to identify and address vulnerabilities that commonly affect older adults with acute illness.

## Key Points

**Question:**

What is the prevalence and cumulative burden associated with geriatric syndromes at hospital admission among older adults, and is the number of syndromes independently associated with 90-day mortality?

**Findings:**

In this cohort study of 2556 adults aged 65 years or older, patients had a median prevalence of 5 geriatric syndromes. A stepwise increase in mortality, from 8.4% (0-2 syndromes) to 47.0% (≥11 syndromes), was observed, and each additional syndrome was associated with a 22% higher adjusted hazard of death within 90 days.

**Meaning:**

These findings suggest that a simple count of geriatric syndromes obtained through multidomain assessment may improve prognostic information and help identify at-risk patients, guide interventions, and inform care planning.

## Teaser

This cohort study investigates the prevalence of geriatric syndromes upon hospital admission of older adults and examines the independent association between number of syndromes and 90-day mortality.

## Introduction

The global aging population is reshaping health care needs, placing considerable demands on systems worldwide.^1^ These pressures are particularly acute in low- and middle-income countries, in which rapid aging is outpacing the development of the necessary infrastructure. As the proportion of individuals aged 65 years or older increases, the health care systems in these countries, which were traditionally designed for younger populations, struggle to adapt to the complex demands of older adults.^2^

Hospitalization further magnifies this complexity because acute illness is superimposed on a background of geriatric syndromes, a group of highly common, age-associated conditions such as frailty, delirium, falls, incontinence, immobility, malnutrition, and functional decline.^3,4,5,6^ Geriatric syndromes can be defined as health conditions with multiple interacting causes, in which deficits across several systems accumulate, leaving older adults susceptible to common stressors.^3,6,7^ Sharing common risk factors and frequently overlapping in presentation, geriatric syndromes defy the traditional disease-based framework and, thus, demand coordinated, multidisciplinary assessment and management.^3,6^ As a result, many older adults are admitted to hospitals with several of these syndromes simultaneously, each compounding the challenges of acute care and amplifying the risk of adverse outcomes, including death.

Despite their interconnectedness, research has tended to consider geriatric syndromes in isolation. Decades of work have clarified the impact of individual conditions, such as frailty and delirium,^8,9^ yet vulnerability rarely appears in isolation. Previous studies have reported that approximately two-thirds of hospitalized older adults live with 1 to 5 geriatric syndromes before or after admission and that 90% of those discharged to skilled nursing facilities have at least 1.^10,11^ However, these reports are limited by their samples and specific health care systems, so the frequency and, more importantly, the prognostic importance of the total syndrome burden remain poorly defined.

Moreover, growing evidence indicates that the accumulation of conditions may better capture baseline health and, thus, more accurately stratify risk than any single marker.^12,13,14^ We therefore designed this study to (1) estimate the prevalence of geriatric syndromes on admission, (2) identify patient- and hospital-level factors associated with a higher cumulative syndrome burden, and (3) evaluate the independent association between the number of syndromes and 90-day all-cause mortality.

## Methods

### Study Design, Setting, and Population

This cohort study uses data from the Creating a Hospital Assessment Network in Geriatrics (CHANGE) study, an international, multicenter, prospective cohort embedded in routine inpatient care designed to support both descriptive and prognostic analyses in hospitalized older adults. Forty-three hospitals (38 in Brazil, 1 in Angola, 1 in Chile, 2 in Colombia, and 1 in Portugal) participated (eTable 1 in Supplement 1). Most sites were recruited through TeleGero, a long-standing tele-education network for geriatric services.^15^ Additional hospitals joined after expressing interest. Ethics approval was obtained at all participating sites, and written informed consent was obtained from participants or their surrogates when capacity was impaired. The study adhered to the Strengthening the Reporting of Observational Studies in Epidemiology (STROBE) reporting guideline^16^ and was registered with the Brazilian Registry of Clinical Trials.^17^

Consecutive patients aged 65 years or older admitted under geriatric teams between June 1, 2022, and December 31, 2023, were assessed for eligibility (eFigure 1 in Supplement 1). Admission to these teams was based on locally defined criteria at each hospital. Across sites, these criteria generally combined an age threshold (usually 60-65 years, occasionally higher) with markers of geriatric complexity (eg, multimorbidity, frailty, dementia, functional dependence, institutionalization) or a request for geriatric consultation or comanagement.

We excluded patients who were terminally ill (defined as having a Clinical Frailty Scale [CFS] score of 9)^18^ and those hospitalized for less than 48 hours. Within 48 hours of admission, a trained rater approached potentially eligible patients at the bedside to describe the study using a plain-language explanation consistent with the approved protocol. Patients were informed that participation was voluntary and would not affect care. Decision-making capacity was assessed clinically; when capacity was impaired, consent was obtained from a representative identified via the medical record or by the attending team. For patients with communication or sensory impairments (eg, hearing or visual loss), raters provided information verbally, verified understanding, and ensured use of personal assistive devices (eg, glasses, hearing aids); a family caregiver could assist with history when necessary. To reduce participant burden, we prioritized the abstraction of routinely collected clinical data (eg, demographics, diagnoses, vital signs, medication lists) and limited direct questioning to items that were not reliably available otherwise.

Data were captured using Research Electronic Data Capture (REDCap) on secure, role-restricted servers with hard-coded fields, real-time completeness checks, and centralized monitoring.^19^ All sites complied with data protection regulations.

### Measurements

Participating sites collaborated closely to standardize data collection procedures, which were developed and refined through a series of structured online meetings facilitated by the central research team (eTable 2 in Supplement 1). These meetings included training on consent procedures, surrogate pathways, instrument administration, and accommodations for individuals with sensory impairments.

Participants were followed up through 3 planned study phases (eFigure 1 and eTable 3 in Supplement 1). Within 48 hours of admission, trained raters documented demographic characteristics, medical history, findings from a comprehensive geriatric assessment, results of the physical examination, and the admitting differential diagnosis. Self-reported race and ethnicity data were included to characterize the diversity of the sample, with categories including Black, White, and other (East Asian, Indigenous, or not declared). Vital signs, oxygen requirement, and level of consciousness were used to calculate illness severity according to the National Early Warning Score (NEWS2).^20^ Chronic diagnoses were used to calculate the Charlson Comorbidity Index, a validated measure of disease burden.^21^ Overall vulnerability was summarized using the 10-Minute Targeted Geriatric Assessment.^22,23^ At discharge, the team recorded length of stay, new diagnoses, complications, procedures, resource use, and disposition. A masked outcomes team then contacted participants or proxies by telephone 30 and 90 days after discharge, making up to 3 attempts and, when necessary, confirming vital status through medical records or public death registries. Center-level characteristics were captured through a standardized questionnaire completed by local principal investigators.

### Outcomes

Primary outcomes were prespecified as (1) the prevalence and within-patient count of 14 geriatric syndromes at admission, including loneliness, dementia, depressive symptoms, sensory impairment, disability, immobility, incontinence, falls, frailty, malnutrition, pressure ulcers, polypharmacy, potentially inappropriate medication use, and delirium (definitions in eTable 4 in Supplement 1), and (2) 90-day all-cause mortality. We selected this panel a priori because each syndrome is common in acute care; can be captured with brief validated instruments; is associated with adverse outcomes; and, taken together, represents physical, cognitive, and psychosocial domains.^18,21,24,25,26,27,28,29,30,31^ Considering the heterogeneity in the literature,^4,32^ the list was intended as pragmatic rather than exhaustive, emphasizing feasibility and bedside operationalization.

### Statistical Analysis

Baseline characteristics were summarized overall and stratified by the cohort’s median number of geriatric syndromes. Given that frailty is sometimes conceptualized as an overarching geriatric syndrome, and frailty assessment may serve as an entry point for identifying other syndromes,^3,6^ the prevalence of other geriatric syndromes was further examined by CFS category. To contrast the association between geriatric syndrome burden and mortality and that of more traditional estimators (eg, comorbidity burden, acute illness severity), 90-day mortality rates were also described across categories of chronic disease counts and NEWS2 scores.

Characteristics associated with the within-patient syndrome count were examined using mixed-effects negative binomial regression, which incorporated random intercepts for state (or province) and hospital to address clustering and account for between-center differences in baseline syndrome burden and case mix. Fixed effects included age, sex, education, race and ethnicity, local Human Development Index (HDI), health care system, recent health care use, and chronic disease count.

Ninety-day all-cause mortality was modeled using mixed-effects Cox proportional hazards regression, with random intercepts for state or province and hospital and a random hospital-level slope for geriatric syndrome count to allow the association between syndrome burden and mortality to vary across centers. Participants without a confirmed 90-day vital status were right censored at the date they were last known alive. Our analytic framework was prognostic rather than causal. Accordingly, the cumulative number of geriatric syndromes at admission was treated as the exposure of interest, and potential confounders (age, sex, education, race and ethnicity, local HDI, health care system, recent health care use, chronic disease count, and NEWS2) were included as adjustment covariates. Coefficients for these covariates were reported to document the adjustment set and to characterize conditional risk gradients and were not intended as estimates of their total causal effects.^33^

Two sensitivity analyses were conducted. First, the cohort was restricted to Brazilian sites to evaluate the robustness of findings within the largest national subset of hospitals. Second, the geriatric syndrome count was recalculated excluding frailty, and frailty was then included as a separate adjustment covariate. This approach allowed the association between syndrome burden and 90-day mortality to be distinguished as being driven primarily by frailty or by the presence of other geriatric syndromes, given the potential overlap between them. Within the primary survival models, the association between the number of geriatric syndromes and 90-day mortality was also evaluated for effect modification by age, sex, and race and ethnicity. Effect modification was summarized on multiplicative and additive scales,^34,35,36^ with adjusted differences in risk illustrated using model-based survival curves.

Data were analyzed from February 1 to November 23, 2025, using Stata, version 18 (StataCorp LLC) and R, version 4.5.2 (R Foundation for Statistical Computing). Two-sided *P* < .05 denoted statistical significance. Because fewer than 2% of key variables were missing, complete-case analysis was applied.

## Results

**Figure 1.**
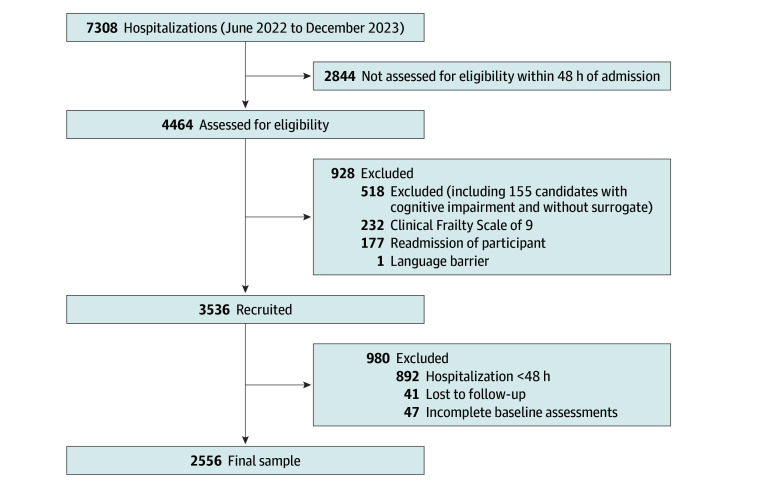
CHANGE Study Inclusion Flow Diagram Of 7308 hospitalizations initially considered, 2844 exceeded recruitment capacity and were not assessed for eligibility. For eligible but nonenrolled patients, screening logs indicated similar aggregate demographics to the enrolled cohort. The majority of included patients were from Brazil (2390 [93.5%]), followed by Colombia (64 [2.5%]), Angola (48 [1.8%]), Portugal (38 [1.5%]), and Chile (16 [1.0%]). Within Brazil, most participants were from the southeastern region (1357 [56.8%]), followed by the northeastern (537 [22.5%]), southern (292 [12.2%]), central-western (137 [5.7%]), and northern (67 [2.8%]) regions.

We enrolled 2556 patients (mean [SD] age, 79 [9] years; 1437 female [56.2%] and 1119 male [43.8%]; 1139 self-reporting as Black [44.6%], 1349 as White [52.8%], and 68 as other [2.7%] race and ethnicity) (Table 1) across 43 hospitals in 5 countries: Angola (1 site [2.3%]), Brazil (38 sites [88.4%]), Chile (1 site [2.3%]), Colombia (2 sites [4.7%]), and Portugal (1 site [2.3%]) (Figure 1; eFigure 2 and eTables 1 and 5 in Supplement 1). Most hospitals (31 [72.1%]) were publicly funded and served patients through government-run health systems. An additional 10 hospitals (23.3%) primarily served patients covered by private insurance, and 2 (4.7%) had a mixed funding model combining public and private payers. Teaching hospitals predominated (39 [90.7%]).

**Table 1.** Sample Characteristics According to the Number of Geriatric Syndromes

Characteristic	Patients, No. (%)			*P* value
	Total (N = 2556)	No. of geriatric syndromes		
		≤5 (n = 1306)	>5 (n = 1250)	
**Sociodemographic**				
Age, mean (SD), y	79 (9)	77 (8)	82 (9)	<.001
Sex				
Female	1437 (56.2)	658 (50.4)	779 (62.3)	<.001
Male	1119 (43.8)	648 (49.6)	471 (37.7)	
Education, median (IQR), y	5 (3-9)	5 (3-10)	4 (3-8)	.001
Race				
Black	1139 (44.6)	635 (48.6)	504 (40.3)	<.001
White	1349 (52.8)	634 (48.5)	715 (57.2)	
Other^a^	68 (2.7)	37 (2.8)	31 (2.5)	
City’s HDI, mean (SD)^b^	0.789 (0.032)	0.787 (0.035)	0.792 (0.029)	<.001
Hospital’s health care system				
Public	1943 (76.0)	1003 (76.8)	940 (75.2)	.55
Private	505 (19.8)	247 (18.9)	258 (20.6)	
Mixed	108 (4.2)	56 (4.3)	52 (4.2)	
Hospital’s level of health care				
Secondary	49 (1.9)	22 (1.7)	27 (2.2)	.38
Tertiary	2507 (98.1)	1284 (98.3)	1223 (97.8)	
Living arrangements				
Alone	363 (14.2)	249 (19.1)	114 (9.1)	<.001
With family	1990 (77.9)	1018 (77.9)	972 (77.8)	
With care partner	109 (4.3)	30 (2.3)	79 (6.3)	
Institutionalized	94 (3.7)	9 (0.7)	85 (6.8)	
**Medical history**				
ED visits or hospitalizations (past 6 mo)				
0	1065 (41.7)	658 (50.4)	407 (32.6)	<.001
≥1 ED visits (no hospitalization)	649 (25.4)	316 (24.2)	333 (26.6)	
≥1 Hospitalization	842 (33.0)	332 (25.4)	510 (40.8)	
Charlson Comorbidity Index, median (IQR)^c^	2 (0-4)	2 (0-3)	2 (1-4)	<.001
Diabetes	954 (37.3)	475 (36.4)	479 (38.3)	.31
Hypertension	1926 (75.4)	973 (74.5)	953 (76.2)	.31
Heart failure	495 (19.4)	228 (17.5)	267 (21.4)	.01
Coronary disease	382 (14.9)	204 (15.6)	178 (14.2)	.33
Previous stroke	401 (15.7)	127 (9.7)	274 (21.9)	<.001
Chronic obstructive pulmonary disease	382 (14.9)	175 (13.4)	207 (16.6)	.03
Chronic liver disease	195 (7.6)	123 (9.4)	72 (5.8)	<.001
Cancer	560 (21.9)	318 (24.3)	242 (19.4)	.002
Osteoarthritis	446 (17.5)	200 (15.3)	246 (19.7)	.004
**Hospitalization**				
Admission				
NEWS2 score, median (IQR)^d^	4 (2-6)	3 (1-5)	5 (3-7)	<.001
10-TaGA score, median (IQR)^e^	0.50 (0.35-0.60)	0.35 (0.25-0.45)	0.56 (0.50-0.65)	<.001
Infection	937 (36.7)	384 (29.4)	553 (44.2)	<.001
Unintentional weight loss	449 (17.6)	211 (16.2)	238 (19.0)	.06
Discharge				
ICU	408 (16.0)	187 (14.3)	221 (17.7)	.02
Surgical procedure	672 (26.3)	384 (29.4)	288 (23.0)	<.001
Palliative care initiation	427 (16.7)	105 (8.0)	322 (25.8)	<.001
Falls during hospitalization^f^				
0	2507 (98.1)	1288 (98.6)	1219 (97.5)	.02
1	43 (1.7)	18 (1.4)	25 (2.0)	
≥2	6 (0.2)	0 (0.0)	6 (0.5)	
Length of stay, median (IQR), d	8 (5-16)	8 (4-14)	10 (6-17)	<.001
In-hospital death	348 (13.6)	87 (6.7)	261 (20.9)	<.001

Abbreviations: 10-TaGA, 10-Minute Targeted Geriatric Assessment; ED, emergency department; HDI, Human Development Index; ICU, intensive care unit; NEWS2, National Early Warning Score 2.

^a^ Included East Asian, Indigenous, and not declared.

^b^ Scale of 0 to 1, with higher values indicating better human development.

^c^ Scale of 0 to 24, with higher values indicating worse burden of comorbidities.

^d^ Scale of 0 to 1, with higher values indicating worse risk of adverse outcomes.

^e^ Scale of 0 to 20, with higher values indicating worse urgency of emergency.

^f^ Document-confirmed events (near-falls not captured); incidence 1.42 per 1000 patient-days.

For patients, formal schooling was limited, with a median duration of 5 years (IQR, 3-9 years) (Table 1). Patient characteristics varied considerably across countries and Brazilian regions, but geriatric vulnerability remained consistently high in all settings (eTable 6 in Supplement 1). The in-hospital mortality rate was 14.0%, and the all-cause mortality rate reached 23.3% 90 days after admission. Vital status at 90 days was unknown for 186 participants (7.3%). These individuals were last known to be alive a median of 10 days after admission (IQR, 4-33 days; range, 2-62 days) and were censored at their last contact in the survival analyses.

**Figure 2.**
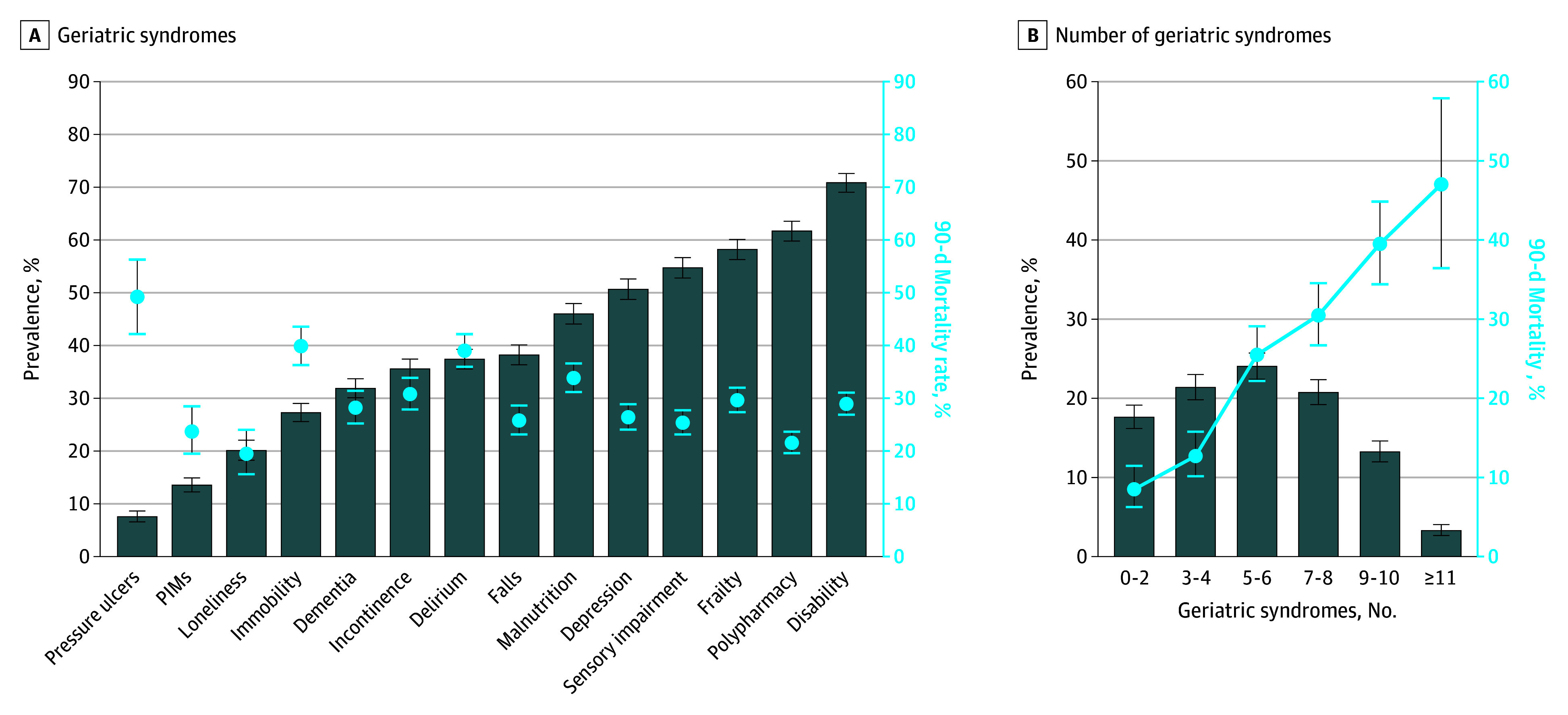
Prevalence of Geriatric Syndromes and 90-Day Mortality in Hospitalized Older Adults and Distribution of Cumulative Conditions A, Prevalence of each syndrome at admission and proportion of patients with that syndrome who died within 90 days. B, Bars indicate the proportion of patients falling into each category of cumulative syndromes, and the line traces the 90-day mortality observed within each category. Error bars represent 95% CIs. PIM indicates potentially inappropriate medication.

Participants accumulated a median of 5 geriatric syndromes (IQR, 3-8 syndromes; mean [SD], 5.5 [2.8] syndromes). The rates for the most prevalent syndromes were 70.8% (95% CI, 69.1%-72.6%) for disability, 61.7% (95% CI, 59.8%-63.6%) for polypharmacy, 58.2% (95% CI, 56.3%-60.1%) for frailty, and 54.7% (95% CI, 52.8%-56.7%) for sensory impairment (Figure 2A). In addition, geriatric syndromes were more frequent among participants who were frail (CFS ≥5) compared with those who were not frail (CFS <5) (eFigure 3 in Supplement 1). The mortality rate at 90 days exceeded 25% for most individual syndromes. Pressure ulcers and delirium were associated with particularly high mortality, with 95 of 193 participants (49.2%) with pressure ulcers and 373 of 956 participants (39.0%) who experienced delirium having died during follow-up.

When geriatric syndromes were examined in aggregate (Figure 2B), prevalence of 5 to 6 syndromes was 24.0% (95% CI, 22.4%-25.7%) and of 7 to 8 syndromes, 20.7% (95% CI, 19.2%-22.3%). Across categories, the mortality rate rose from 8.4% (95% CI, 6.2%-11.4%) for 0 to 2 syndromes to 12.7% (95% CI, 10.1%-15.7%) for 3 to 4 syndromes, 25.4% (95% CI, 22.2%-29.1%) for 5 to 6 syndromes, 30.4% (95% CI, 26.7%-34.5%) for 7 to 8 syndromes, 39.5% (95% CI, 34.4%-44.8%) for 9 to 10 syndromes, and 47.0% (95% CI, 36.4%-57.9%) for 11 or more syndromes. Participants with more than 5 geriatric syndromes carried 33.0% (95% CI, 30.5%-35.7%) of the 90-day death rate compared with 5 of fewer syndromes (14.0% [95% CI, 12.2%-16.0%]). Mortality rate differences across chronic disease categories were smaller and did not follow a consistent pattern (0-1 conditions, 27.4% [95% CI, 23.4%-31.8%]; 2-3 conditions, 21.8% [95% CI, 19.5%-24.4%]; ≥4 conditions, 23.1% [95% CI, 20.7%-25.8%]). In contrast, mortality rates increased across NEWS2 categories, rising from 16.1% (95% CI, 14.3%-18.1%) to 29.3% (95% CI, 25.7%-33.2%) to 36.9% (95% CI, 32.9%-41.2%) for scores of 0 to 4, 5 to 6, and 7 or higher, respectively.

Our multivariable analyses showed that the burden of geriatric syndromes rose steadily with advancing age (Table 2). Each additional 5 years of age was independently associated with an increase in the expected number of syndromes (incidence rate ratio [IRR], 1.08 [95% CI, 1.07-1.10]) (eFigure 4 in Supplement 1). Syndrome burden was also associated with female sex (IRR, 1.07 [95% CI, 1.03-1.11]), lower education (IRR, 0.98 [95% CI, 0.96-0.99]), greater multimorbidity (IRR, 1.03 [95% CI, 1.02-1.04]), and recent emergency department visits (IRR, 1.18 [95% CI, 1.13-1.24]) or hospitalizations (IRR, 1.33 [95% CI, 1.27-1.39]).

**Table 2.** Associations of Patient and System Characteristics With Geriatric Syndrome Count and Geriatric Syndrome Count With 90-Day All-Cause Mortality in Hospitalized Older Adults (N = 2556)

Variable	Unadjusted IRR or HR (95% CI)^a^	*P* value	Adjusted IRR or HR (95% CI)^a^	*P* value
**Association of participant or system characteristics with No. of geriatric syndromes**				
Age (per 5 y)	1.08 (1.07-1.20)	<.001	1.08 (1.07-1.10)	<.001
Sex				
Female	1.11 (1.07-1.15)	<.001	1.07 (1.03-1.11)	<.001
Male	1 [Reference]	NA	1 [Reference]	NA
Race and ethnicity				
Black	0.98 (0.93-1.02)	.29	0.99 (0.95-1.03)	.61
White or other	1 [Reference]	NA	1 [Reference]	NA
Education (per 4 y)	0.95 (0.93-0.97)	<.001	0.98 (0.96-0.99)	.007
HDI (per 0.1 unit)	1.20 (0.94-1.53)	.14	1.13 (0.96-1.33)	.16
Health care system				
Private or mixed	1 [Reference]	NA	1 [Reference]	NA
Public	1.06 (0.91-1.23)	.44	1.11 (0.98-1.25)	.09
ED visits or hospitalizations (past 6 mo)				
0	1 [Reference]	NA	1 [Reference]	NA
≥1 ED visits (no hospitalizations)	1.21 (1.15-1.27)	<.001	1.18 (1.13-1.24)	<.001
≥1 Hospitalizations	1.34 (1.28-1.40)	<.001	1.33 (1.27-1.39)	<.001
No. of chronic diseases^b^	1.04 (1.03-1.05)	<.001	1.03 (1.02-1.04)	<.001
**Association of No. of geriatric syndromes with 90-d mortality^c^**				
No. of geriatric syndromes	1.24 (1.20-1.28)	<.001	1.22 (1.15-1.30)	<.001
Age (per 5 y)	1.16 (1.10-1.22)	<.001	1.08 (1.03-1.14)	.004
Sex				
Male	1 [Reference]	NA	1 [Reference]	NA
Female	0.84 (0.71-0.98)	.03	0.73 (0.61-0.86)	<.001
Race				
Black	0.94 (0.78-1.13)	.47	1.00 (0.83-1.20)	.99
White or other	1 [Reference]	NA	1 [Reference]	NA
Education (per 4 y)	0.94 (0.86-1.01)	.09	1.04 (0.96-1.13)	.35
HDI (per 0.1 unit)	1.36 (0.76-2.43)	.30	1.09 (0.67-1.79)	.72
Health care system				
Private or mixed	1 [Reference]	NA	1 [Reference]	NA
Public health system	2.37 (1.64-3.42)	<.001	3.04 (2.00-4.60)	<.001
ED visits or hospitalizations (past 6 mo)				
0	1 [Reference]	NA	1 [Reference]	NA
≥1 ED visits (no hospitalizations)	1.24 (1.01-1.53)	.04	1.03 (0.83-1.28)	.81
≥1 Hospitalizations	1.51 (1.24-1.84)	<.001	1.19 (0.97-1.46)	.11
No. of chronic diseases^b^	0.96 (0.92-1.00)	.07	0.91 (0.88-0.96)	<.001
NEWS2 score	1.18 (1.15-1.21)	<.001	1.12 (1.08-1.15)	<.001

Abbreviations: HDI, Human Development Index; HR, hazard ratio; IRR, incidence rate ratio; NA, not applicable; NEWS2, National Early Warning Score 2.

^a^ The IRRs (calculated from mixed-effects negative binomial regression models with random intercepts at the state or province and hospital levels) are shown for associations of patient and system characteristics with number of geriatric syndromes, and HRs (calculated from Cox proportional hazards models with random intercepts at the state or province and hospital levels) are shown for associations of number of geriatric syndromes with 90-day mortality. The multivariable models adjust for all listed covariates.

^b^ Number of chronic diseases among stroke, anxiety, asthma, transient ischemic attack, depression, diabetes, chronic kidney disease, connective tissue disease, chronic obstructive pulmonary disease, atrial fibrillation, liver disease, hypertension, myocardial infarction, vascular disease, heart failure, coronary artery disease, hematologic cancer, other cancers, osteoarthritis, osteoporosis, HIV, and peptic ulcer disease.

^c^ Coefficients for covariates other than the geriatric syndrome count are shown to characterize conditional risk gradients and to document the adjustment set. Because they are estimated from models that condition on other factors, they should not be interpreted as estimates of the total causal effects of those variables.^33^

**Figure 3.**
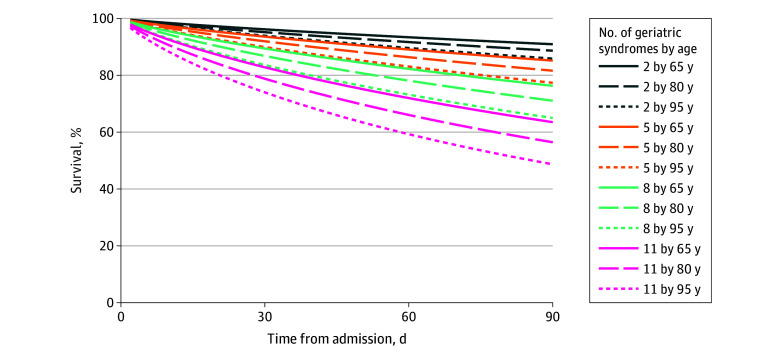
Survival Probabilities Stratified by Number of Geriatric Syndromes and Age in Hospitalized Older Adults Curves were generated from the fully adjusted mixed-effects Cox proportional hazards model.

We found that the number of geriatric syndromes was independently associated with mortality (Table 2). Each additional syndrome was associated with an increased hazard of death within 90 days (adjusted hazard ratio [HR], 1.22 [95% CI, 1.15-1.30]). Within the same multivariable model, older age (adjusted HR, 1.08 [95% CI, 1.03-1.14]), care in publicly funded hospitals (adjusted HR, 3.04 [95% CI, 2.00-4.60]), and NEWS2 scores (adjusted HR, 1.15 [95% CI, 1.08-1.15]) showed associations with increased mortality. Findings were largely confirmed when analyses were limited to Brazilian hospitals (eTable 7 in Supplement 1). Moreover, when frailty was excluded from the geriatric syndrome count and modeled as a separate covariate, the revised syndrome count remained associated with 90-day mortality (HR, 1.23 [95% CI, 1.15-1.31]). Frailty itself had an estimated HR of 1.20 (95% CI, 0.96-1.50). Finally, our effect modification analyses indicated that age altered the strength of the association between syndrome burden and survival, with a steeper mortality gradient at older ages (Figure 3; eTable 8 in Supplement 1).

## Discussion

This cohort study provided a detailed profile of geriatric syndromes in hospitalized older adults from diverse centers across 5 countries, predominantly low- and middle-income settings. Our findings revealed a strikingly high prevalence of geriatric syndromes, with nearly all patients presenting with at least 1 such condition. The cumulative burden of geriatric syndromes was substantial, with a median of 5 syndromes per patient, underscoring the complex health challenges faced by this population. Notably, the risk of death was associated with the number of geriatric syndromes, with each additional syndrome associated with a 22% increase in the hazard of death within 90 days of admission. Our findings also suggest that the prognostic contribution of geriatric syndromes may not be simply a proxy for chronic disease burden or illness acuity and that multidomain evaluations may provide prognostic information beyond traditional markers in hospital settings.

Previous work from high-income regions has also documented substantial syndrome counts.^11,37,38,39,40^ In a study of 6 Dutch hospitals, van Seben et al^38^ found a median of 5 syndromes at admission, with 62% and 55% of patients experiencing activities of daily living disability and immobility, respectively. In 4 Australian public hospitals, Mudge et al^39^ reported activities of daily living disability in 22%, recent falls in 37%, and malnutrition in 40% of patients. Analyses of US Medicare transfers to skilled nursing facilities have shown comparably high rates of incontinence (39%), cognitive impairment (25%), and pressure ulcers (15%).^11^ By drawing on data from 43 hospitals, most in low- and middle-income settings, our study extends this literature, showing even higher prevalence figures and underscoring that geriatric vulnerability is at least as pronounced, and possibly amplified, in resource-constrained settings. These data also complement community-based surveys from the Global South,^40^ and argue for tailored inpatient strategies that acknowledge both the breadth and cumulative impact of geriatric syndromes.

We verified a pronounced survival gradient among funding models: after adjustment, treatment in a publicly financed hospital conferred 3 times the 90-day mortality risk observed in private or mixed systems. This disparity is consistent with prior work showing that hospitals serving predominantly populations with lower socioeconomic status have higher in-hospital and postdischarge mortality rates among older adults, even after accounting for comorbidity burden and illness severity.^41,42,43^ Authors have attributed the excess risk to a confluence of structural and social factors, including less robust infrastructure; leaner staffing ratios; slower escalation of care for time-sensitive conditions, such as sepsis; and the cumulative health disadvantages of patients with lower income and less education.^44,45^

In our study, each additional geriatric syndrome increased the adjusted hazard of 90-day death by more than one-fifth, with a steeper gradient at older ages. Prior work often examined single domains (eg, delirium, frailty, disability) and associated them with adverse outcomes,^29,46,47^ while a population-based cohort study in Stockholm, Sweden, treated geriatric syndromes dichotomously (any vs none) and observed persistently higher inpatient and outpatient health care use and polypharmacy over 4 years.^32^ By quantifying 14 syndromes concurrently and modeling their accumulation, we extend that evidence and highlight the prognostic value of considering them in concert rather than in isolation. Capturing this multidimensional risk requires assessments that reach beyond disease-centric measures to include functional, cognitive, psychological, and social domains. Even with the increasing use of natural language processing tools to mine electronic health records for frailty and prognosis, accuracy remains constrained by the sparse routine documentation of many geriatric domains.

The Comprehensive Geriatric Assessment (CGA) was designed precisely for this purpose. It synthesizes information on mobility, cognition, mood, nutrition, medication use, and social support to guide therapeutic, rehabilitative, and long-term-care decisions.^3,6,11,48,49^ Randomized and observational studies have shown that CGA improves functional outcomes, increases the likelihood of discharge home, and enhances survival.^50,51,52^ Accordingly, recent international guidance recommends routine CGA in hospitalized older adults to detect geriatric syndromes and inform treatment.^53,54^ Despite this evidence, implementation remains uneven, especially in resource-limited settings in which time, staffing, and infrastructure are critical constraints. Prognostic instruments, such as the Multidimensional Prognostic Index, have been shown to improve risk stratification and are now available in various formats, including telephone, brief, and self-administered versions, as well as smart device application-based calculators. However, these instruments rely on structured multidomain inputs (eg, function, cognition, nutrition) that are not consistently captured at admission in many hospitals.^5,22,47,55,56,57,58,59,60^ Our findings reinforce the clinical value of CGA-like approaches and the need to develop streamlined, context-appropriate tools to identify and mitigate geriatric vulnerability in hospitals worldwide.^23,61^

Most geriatric syndromes are rarely perceived as diagnostic entities by clinicians outside geriatric medicine. However, we found that quantifying their cumulative burden may provide prognostic information that complements age, number of chronic conditions, and traditional acute care measures. Simply incorporating this count into routine hospital assessments may improve prognostic accuracy in older adults with acute illness, prompt timely palliative or rehabilitative care, and support more informed discussions with patients and families about likely outcomes. Moreover, documenting factors associated with syndrome accumulation could guide hospital-wide quality improvement programs aligned with the Age-Friendly Health Systems framework, which emphasizes mobility, mentation, medication safety, and attention to what matters most to older adults.^62^

### Strengths and Limitations

This investigation enrolled more than 2500 patients in a prospective design that spanned 43 hospitals on 3 continents, most of them in low- and middle-income settings. The sample size afforded robust estimates of both syndrome prevalence and short-term mortality, while the multisite framework captured practice patterns across a range of geographic, socioeconomic, and organizational contexts. All centers applied the same validated instruments, permitting a systematic appraisal of 14 geriatric syndromes (far broader than the domain-specific assessments that dominate the literature) and ensuring internal consistency in data collection. Notably, low- and middle-income countries now account for most of the world’s rapidly expanding older population.^63^ To our knowledge, no large, prospective, multicenter investigation has quantified the full spectrum of geriatric syndromes at the bedside or examined their joint association with mortality in this context.

The study’s limitations must also be considered. First, participation was based on center interest rather than population sampling, and nearly 9 in 10 hospitals were located in Brazil; therefore, external validity for other regions warrants confirmation. Second, participants were recruited from patients under geriatric care, who tended to have more clinical complexity, although this is also the group in whom geriatric assessment and management decisions are most directly relevant. Third, recruitment capacity varied across sites, resulting in some eligible admissions not being screened. Available data suggested that these nonenrolled patients were demographically similar to our participants and that strong systematic bias was unlikely. Nevertheless, some selection by unmeasured factors cannot be excluded. Fourth, our multicenter design naturally raised questions about heterogeneity in both baseline risk and the association between geriatric syndrome burden and mortality. To account for heterogeneity, we fitted mixed-effects proportional hazards models with random intercepts for state or province and hospital and a hospital-level random slope for the syndrome count, adjusting for possible confounders. However, we did not attempt to estimate the causal effect of geriatric syndromes or their total number on mortality, which would have required a counterfactual framework and additional design and modeling assumptions beyond the scope of this study.^64^ Future work could build on this cohort to address causal questions using approaches such as causal meta-analysis, transportability methods, or target trial emulation to better account for heterogeneity and bias across settings.^65,66^ Fifth, our operational list of syndromes was not exhaustive, and several items potentially overlapped. By design, we used an unweighted count to prioritize feasibility and transportability, foregoing syndrome-specific weights or cluster-based reclassifications. Future analyses could incorporate co-occurrence mapping and clustering to identify high-risk profiles that are actionable at the ward level. Finally, patients or members of the public were not involved in the development of this study, which may have limited the extent to which our outcomes and data collection priorities fully represent patient-valued goals.^67^ Moving forward, we are committed to incorporating patient and public involvement and engagement in our work.

## Conclusions

This cohort study of 2556 hospitalized older adults found that geriatric syndromes were ubiquitous in acute care and that their cumulative burden was independently associated with 90-day mortality. The findings point to the clinical value of comprehensive, multidomain assessments that quantify functional, cognitive, psychosocial, and medical vulnerabilities rather than rely solely on disease-centric measures. Future implementation research should focus on pragmatic strategies to embed such assessments into routine hospital workflows sustainably, particularly in resource-constrained settings in which the need is greatest. Such integration is a necessary step toward delivering age-appropriate, outcome-oriented acute care.

## Notes

Supplement 1. **eFigure 1.** Timeline of Participant Assessments and Follow-Up in the CHANGE Study **eFigure 2.** Geographic Distribution of Participants and Median Geriatric Syndromes Across Cities in the CHANGE Study **eFigure 3.** Prevalence of Geriatric Syndromes According to Frailty Status at Admission **eFigure 4.** Age-Related Estimated Probabilities of Geriatric Syndromes in Hospitalized Older Adults **eTable 1.** Distribution of Participating Centers in the CHANGE Study by Country and Region **eTable 2.** Standardization of Data Collection Procedures Across CHANGE Study Sites **eTable 3.** Summary of Assessments Conducted at Admission, Discharge, and Follow-Up in the CHANGE Study **eTable 4.** Definitions and Assessment Tools for Geriatric Syndromes **eTable 5.** Characteristics of Participating Centers in the CHANGE Study by Country and Region **eTable 6.** Baseline Sociodemographic, Health System, and Clinical Case Mix of Hospitalized Older Adults by Country and Region **eTable 7.** Multivariable Mixed-Effects Models Relating Patient and System Characteristics to Cumulative Geriatric Syndrome Burden and 90-Day All-Cause Mortality in Brazilian Hospitalized older Adults **eTable 8.** Modification of the Association of Geriatric Syndrome Burden With 90-Day Mortality by Age

Supplement 2.
Nonauthor Collaborators

Supplement 3.
Data Sharing Statement
