# Supplementary material for: Geriatric Syndromes and Mortality Among Hospitalized Older Adults
Source: JAMA Netw Open. 2026 Jan 27;9(1):e2555740. doi: 10.1001/jamanetworkopen.2025.55740 (PMC12848630; doi:10.1001/jamanetworkopen.2025.55740)
Supplement: Supplement 1. — eFigure 1. Timeline of Participant Assessments and Follow-Up in the CHANGE Study eFigure 2. Geographic Distribution of Participants and Median Geriatric Syndromes Across Cities in the CHANGE Study eFigure 3. Prevalence of Geriatric Syndromes According to Frailty Status at Admission eFigure 4. Age-Related Estimated Probabilities of Geriatric Syndromes in Hospitalized Older Adults eTable 1. Distribution of Participating Centers in the CHANGE Study by Country and Region eTable 2. Standardization of Data Collection Procedures Across CHANGE Study Sites eTable 3. Summary of Assessments Conducted at Admission, Discharge, and Follow-Up in the CHANGE Study eTable 4. Definitions and Assessment Tools for Geriatric Syndromes eTable 5. Characteristics of Participating Centers in the CHANGE Study by Country and Region eTable 6. Baseline Sociodemographic, Health System, and Clinical Case Mix of Hospitalized Older Adults by Country and Region eTable 7. Multivariable Mixed-Effects Models Relating Patient and System Characteristics to Cumulative Geriatric Syndrome Burden and 90-Day All-Cause Mortality in Brazilian Hospitalized older Adults eTable 8. Modification of the Association of Geriatric Syndrome Burden With 90-Day Mortality by Age [file jamanetwopen-e2555740-s001.pdf]

Supplemental Online Content

Avelino-Silva TJ, Roma MFB, Dutra AF, et al.; CHANGE Study Group. Geriatric syndromes and mortality among hospitalized older adults. *JAMA Netw Open*. 2026;9(1):e2555740. doi:10.1001/jamanetworkopen.2025.55740

eFigure 1. Timeline of Participant Assessments and Follow-Up in the CHANGE Study

eFigure 2. Geographic Distribution of Participants and Median Geriatric Syndromes Across Cities in the CHANGE Study

eFigure 3. Prevalence of Geriatric Syndromes According to Frailty Status at Admission

eFigure 4. Age-Related Estimated Probabilities of Geriatric Syndromes in Hospitalized Older Adults

eTable 1. Distribution of Participating Centers in the CHANGE Study by Country and Region

eTable 2. Standardization of Data Collection Procedures Across CHANGE Study Sites

eTable 3. Summary of Assessments Conducted at Admission, Discharge, and Follow-Up in the CHANGE Study

eTable 4. Definitions and Assessment Tools for Geriatric Syndromes

eTable 5. Characteristics of Participating Centers in the CHANGE Study by Country and Region

eTable 6. Baseline Sociodemographic, Health System, and Clinical Case Mix of Hospitalized Older Adults by Country and Region

eTable 7. Multivariable Mixed-Effects Models Relating Patient and System Characteristics to Cumulative Geriatric Syndrome Burden and 90-Day All-Cause Mortality in Brazilian Hospitalized older Adults

eTable 8. Modification of the Association of Geriatric Syndrome Burden With 90-Day Mortality by Age

This supplemental material has been provided by the authors to give readers additional information about their work.

© 2026 Avelino-Silva TJ et al. *JAMA Network Open*.

eFigure 1. Timeline of Participant Assessments and Follow-Up in the CHANGE Study

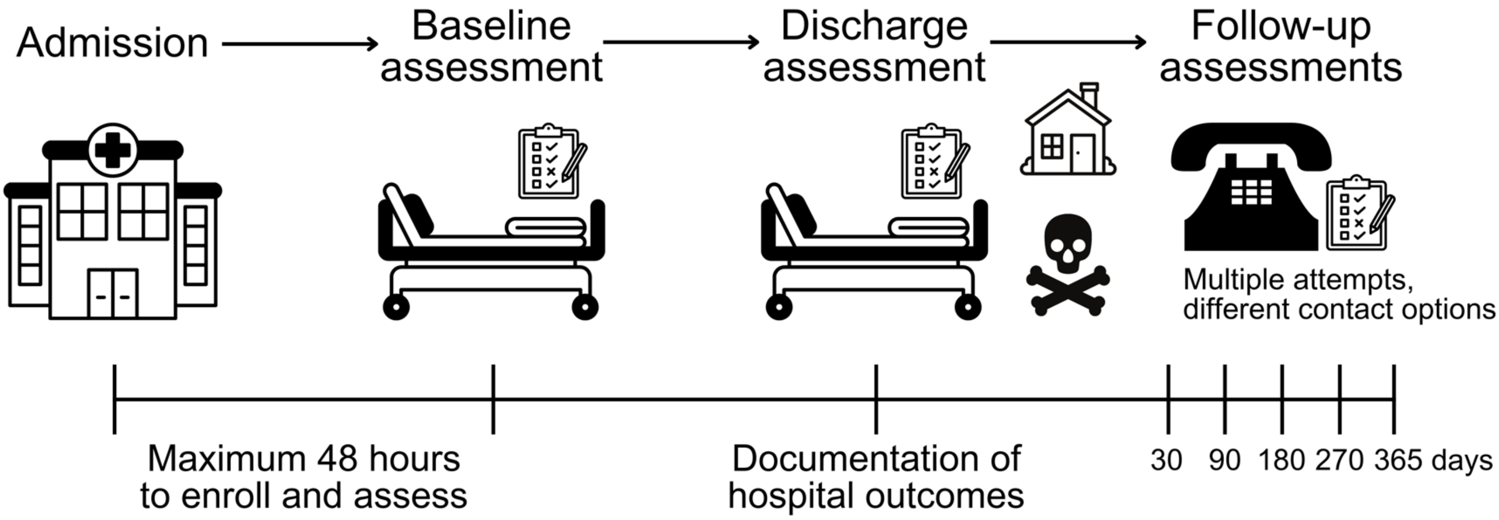

Patients were enrolled and received a standardized baseline assessment within 48 hours of admission; a second assessment at discharge recorded clinical course and disposition. Vital status and other postdischarge data were then collected by structured telephone interviews at 30, 90, 180, 270, and 365 days, with repeated contact attempts and use of proxies when needed. The analyses in this manuscript are restricted to the 90-day follow-up.

© 2026 Avelino-Silva TJ et al. *JAMA Network Open*.

eFigure 2: Geographic Distribution of Participants and Median Geriatric Syndromes Across Cities in the CHANGE Study

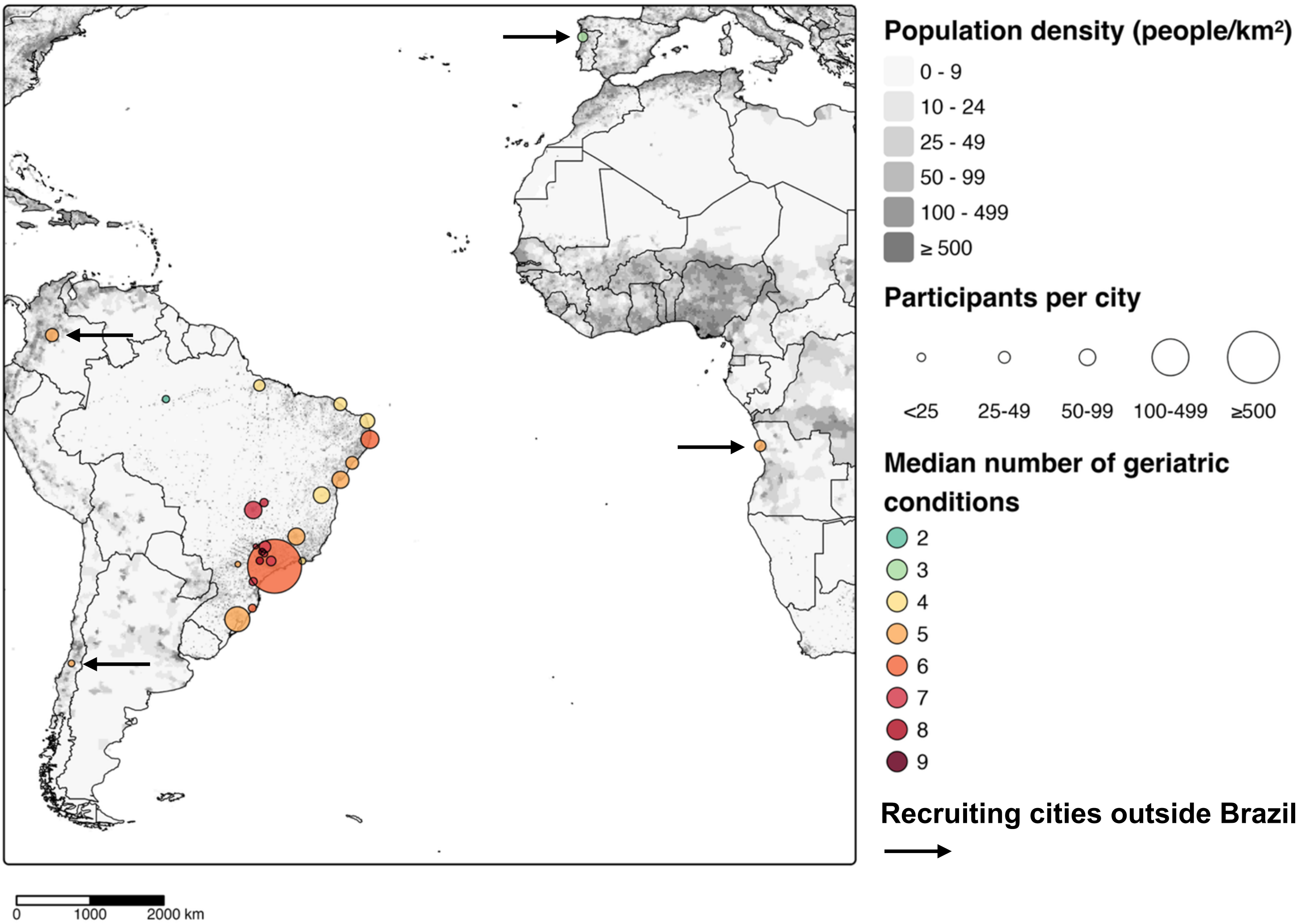

Gridded Population of the World, Version 4 (GPWv4): Population Density, Revision 11. Palisades, NY: NASA Socioeconomic Data and Applications Center (SEDAC). https://doi.org/10.7927/H49C6VHW. Accessed February 24, 2025.

© 2026 Avelino-Silva TJ et al. *JAMA Network Open*.

eFigure 3. Prevalence of Geriatric Syndromes According to Frailty Status at Admission (N=2,556).

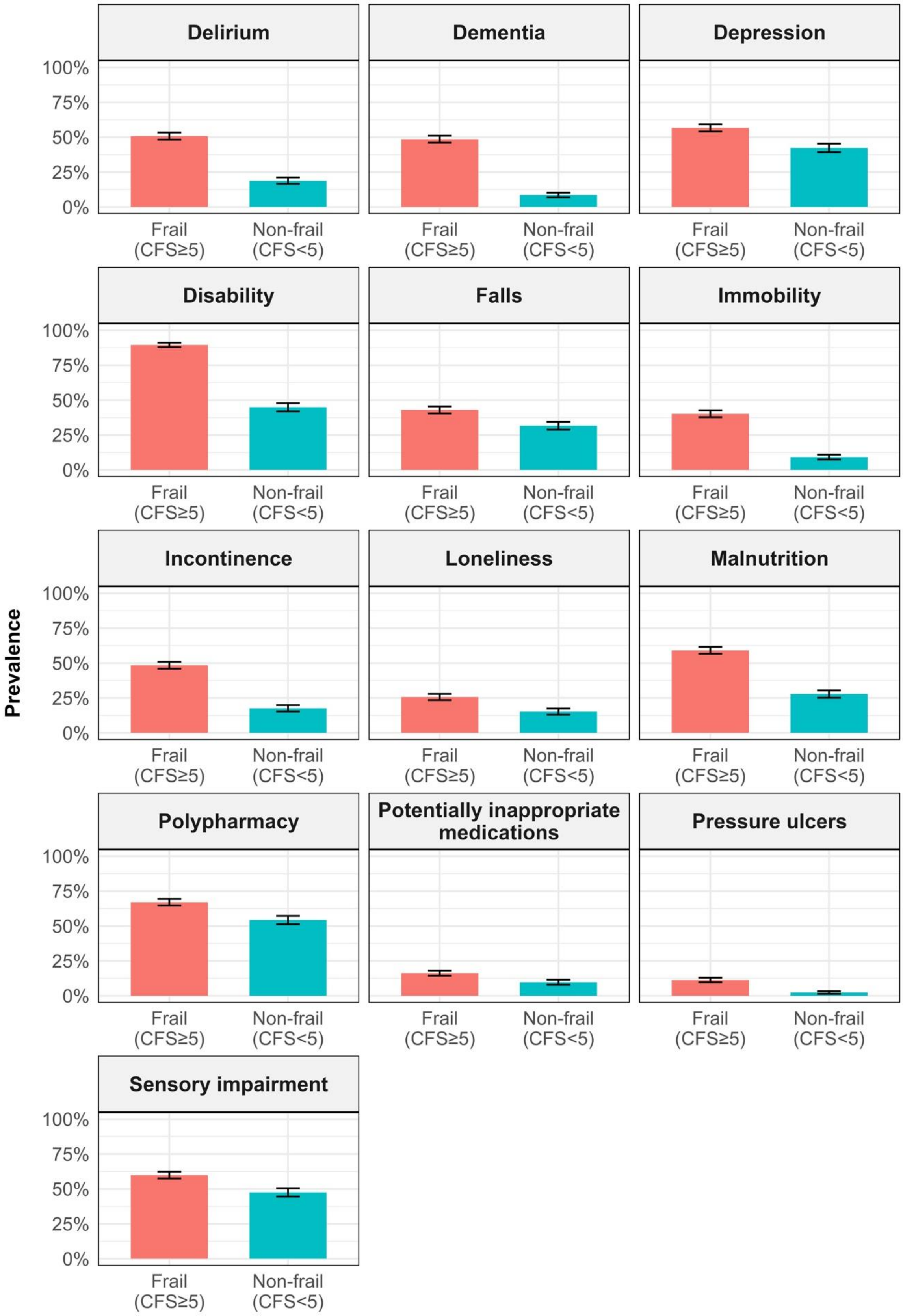

Bar charts show the prevalence (with 95% confidence intervals) of each geriatric syndrome among participants classified as frail (Clinical Frailty Scale [CFS] ≥5) and non-frail (CFS <5) at hospital admission. For every syndrome displayed, prevalence was higher in frail than in non-frail participants (all p<0.001).

© 2026 Avelino-Silva TJ et al. *JAMA Network Open*.

eFigure 4. Age-Related Estimated Probabilities of Geriatric Syndromes in Hospitalized Older Adults

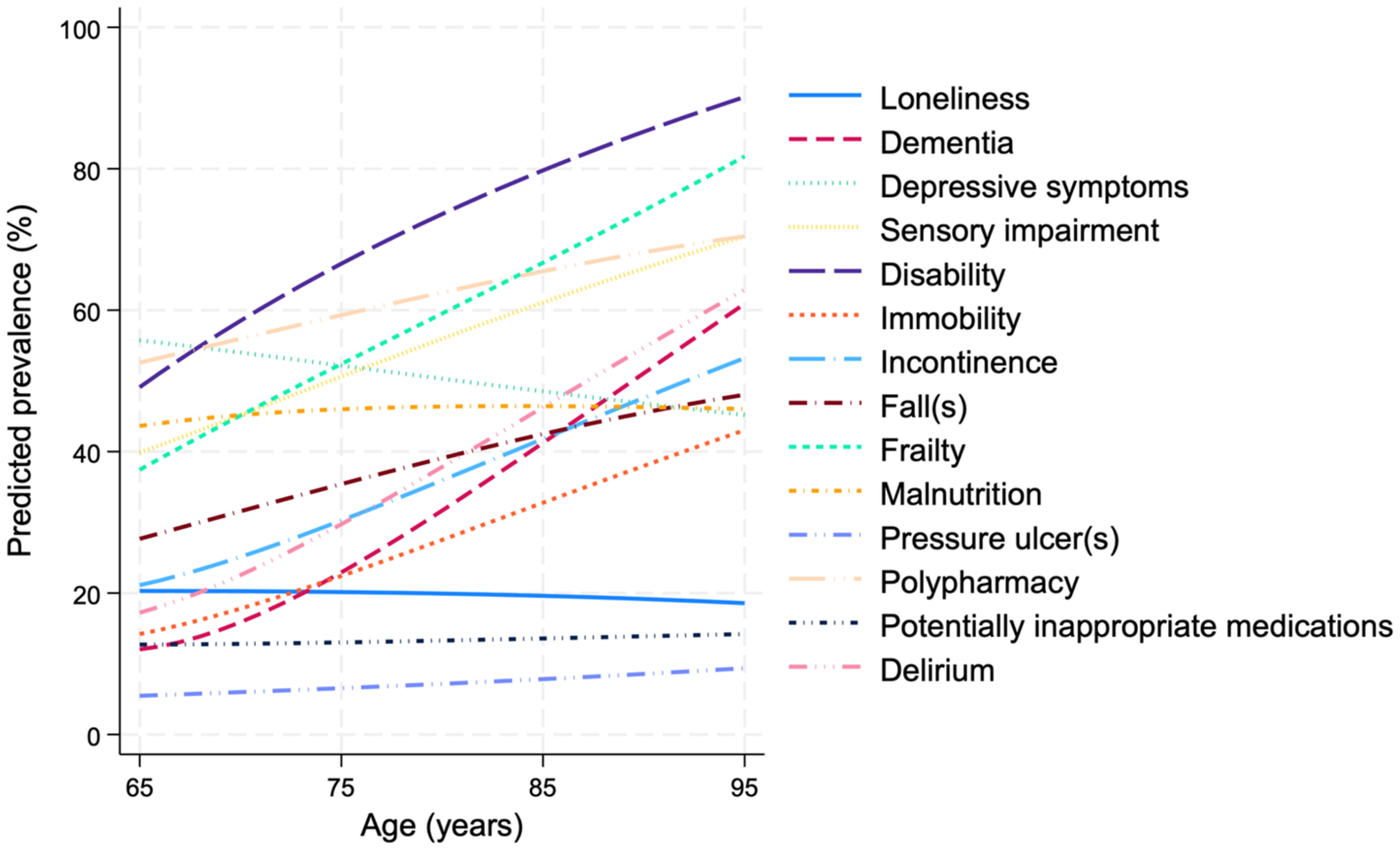

Age-specific predicted prevalence of 14 geriatric syndromes obtained from multilevel logistic regression models with random intercepts for state and hospital. Most conditions rise monotonically from age 65 to 95, illustrating the progressive accumulation of syndromes with advancing age.

© 2026 Avelino-Silva TJ et al. *JAMA Network Open*.

eTable 1. Distribution of Participating Centers in the CHANGE Study by Country and Region

Participating center (N=43)	n	%
**Angola**		
Clínica Girassol, Luanda, Luanda	48	2
**Chile**		
Hospital Regional de Talca Dr. César Garavagno Burotto, Talca, Maule	16	1
**Colombia**		
Hospital Universitario Mayor Méderi, Bogotá, DC	54	2
Hospital Universitario San Ignacio, Bogotá, DC	10	0
**Brazil**		
South		
Hospital Nossa Senhora das Graças, Curitiba, PR	24	1
Hospital Universitário da Universidade Estadual de Londrina, Londrina, PR	12	0
Hospital de Clínicas de Porto Alegre, Porto Alegre, RS	233	9
Hospital São José, Criciúma, SC	23	1
Southeast		
Hospital das Clínicas da Universidade Federal de Minas Gerais, Belo Horizonte, MG	108	4
Hospital Universitário Pedro Ernesto, Rio de Janeiro, RJ	17	1
A. C. Camargo Cancer Center, São Paulo, SP	19	1
Beneficência Portuguesa de São Paulo, São Paulo, SP	23	1
Hospital Central da Irmandade da Santa Casa de Misericórdia de São Paulo, São Paulo, SP	22	1
Hospital das Clínicas da Faculdade de Medicina de Botucatu, Botucatu, SP	20	1
Hospital das Clínicas da Faculdade de Medicina de Ribeirão Preto, Ribeirão Preto, SP	50	2
Hospital das Clínicas da Faculdade de Medicina da USP, São Paulo, SP	415	16
Hospital de Clínicas - UNICAMP, Campinas, SP	34	1
Hospital do Coração, São Paulo, SP	20	1
Hospital Emílio Carlos - Centro Universitário Padre Albino, Catanduva, SP	9	0
Hospital Geriátrico e de Convalescentes Dom Pedro II, São Paulo, SP	15	1
Hospital Israelita Albert Einstein, São Paulo, SP	17	1
Hospital São Camilo, São Paulo, SP	20	1
Hospital São Paulo - UNIFESP, São Paulo, SP	22	1
Hospital Sírio Libanês, São Paulo, SP	207	8
Hospital Universitário da Universidade Federal de São Carlos, São Carlos, SP	15	1
Instituto do Câncer do Estado de São Paulo, São Paulo, SP	307	12
Santa Casa de Araraquara, Araraquara, SP	17	1
Central-West		
Hospital de Urgências de Goiás, Goiânia, GO	112	4
Hospital Universitário de Brasília, Brasília, DF	25	1
Northeast		
Hospital Geral de Vitória da Conquista, Vitória da Conquista, BA	102	4
Hospital Santo Antônio - Obras Sociais Irmã Dulce, Salvador, BA	86	3
Hospital Universitário Professor Edgard Santos, Salvador, BA	22	1
Hospital Geral Dr. César Cals de Oliveira, Fortaleza, CE	63	2
Hospital das Clínicas da Universidade Federal de Pernambuco, Recife, PE	19	1
Instituto de Medicina Integral Prof. Fernando Figueira, Recife, PE	13	1
Real Hospital Português de Beneficência, Recife, PE	89	3
Hospital do Coração de Natal, Natal, RN	54	2
Hospital Universitário Onofre Lopes, Natal, RN	28	1
Hospital Universitário da Universidade Federal de Sergipe, Aracaju, SE	61	2
North		
Hospital Universitário Getúlio Vargas, Manaus, AM	19	1
Hospital Regional Jean Bitar, Belém, PA	42	2
Hospital Universitário João de Barros Barreto, Belém, PA	6	0
**Portugal**		
Hospital Lusíadas Porto, Porto	38	1
**Total**	**2,556**	**100**

N: number of participating centers; n: number of patients enrolled at each participating center; %: percentage of total enrollment across all centers (N=2,556); PR: Paraná; RS: Rio Grande do Sul; SC: Santa Catarina; MG: Minas Gerais; RJ: Rio de Janeiro; SP: São Paulo; GO: Goiás; DF: Distrito Federal; BA: Bahia; CE: Ceará; PE: Pernambuco; RN: Rio Grande do Norte; SE: Sergipe; AM: Amazonas; PA: Pará

© 2026 Avelino-Silva TJ et al. *JAMA Network Open*.

eTable 2. Standardization of Data Collection Procedures Across CHANGE Study Sites

Process component	Key actions	Frequency / timing	Responsible parties	Output / purpose
**Protocol co-development**	• Structured online meetings to draft and refine assessment protocol<br>• Literature review to select validated instruments	Monthly (pre-launch)	Central research team + site PIs	Harmonized, evidence-based assessment protocol
**Training & capacity building**	• Live virtual workshops on study workflow and instrument use<br>• Instructional videos and written guides circulated to all staff	Once after protocol finalization; materials available on-demand	Central team (trainers)	Consistent, site-wide application of evaluation methods
**REDCap implementation**	• Creation of standardized electronic case-report forms (eCRFs)<br>• Site-specific user accounts with audit trails	Before first patient enrollment	Data-management subteam	Real-time data entry, centralized monitoring, automated completeness checks
**REDCap user training**	• Hands-on sessions covering data entry, query resolution, and error avoidance	At site onboarding	Data-management subteam	Accurate, high-quality data capture
**Ongoing quality assurance**	• Monthly cross-site teleconferences to review recruitment progress, troubleshoot issues, and update SOPs<br>• Feedback loop on data discrepancies and missing fields	Monthly (throughout enrollment)	Central research team + site coordinators	Continuous protocol adherence and remediation of data-quality gaps
**Continuous education**	• Targeted refreshers (e.g., short videos, tip-sheets) addressing common errors identified in QA reviews	As needed (triggered by QA findings)	Central research team	Sustained performance improvement

PIs: Principal Investigators; QA: Quality assurance; SOPs: Standard Operating Procedures.

© 2026 Avelino-Silva TJ et al. *JAMA Network Open*.

eTable 3. Summary of Assessments Conducted At Admission, Discharge, and Follow-Up in the CHANGE Study

ADMISSION

Demographics
- Age
- Sex
- Gender
- Self-reported race/ethnicity
- Marital status
- Education

Additional informant summary
- Relationship with participant
- Care partner
- Frequency of interaction

Medical history
- Diagnoses
- CHARLSON COMORBIDITY INDEX
- Medications
- Smoking
- Alcohol consumption
- Vaccination status
- Physical activity
- UCLA 3-ITEM LONELINESS SCALE
- Memory complaints
- CLINICAL DEMENTIA RATING (CDR)

10-MINUTE TARGETED GERIATRIC ASSESSMENT (10-TaGA)
- Living arrangement
- Social support
- History of emergency department visits and hospitalizations (previous six months)
- Falls (previous 12 months)
- Number of medications
- Activities of daily living (ADLs) (current and one month before admission)
- CONFUSION ASSESSMENT METHOD (CAM)
- 10-POINT COGNITIVE SCREENER (10-CS)
- Self-assessed health status
- 4 QUESTIONS GERIATRIC DEPRESSION SCALE (GDS-4)
- Body mass index
- Weight loss history
- Gait speed

MULTIDIMENSIONAL PROGNOSIS INDEX (MPI)
(in addition to living arrangement, number of medications, and activities of daily living)
- Instrumental activities of daily living (IADLs)

© 2026 Avelino-Silva TJ et al. *JAMA Network Open*.

- SHORT PORTABLE MENTAL STATUS QUESTIONNAIRE (SPMSQ)
- EXTON-SMITH SCALE (ESS)
- CUMULATIVE ILLNESS RATING SCALE (CIRS)
- MINI-NUTRITIONAL ASSESSMENT (MNA)

Frailty assessment
- FRAIL SCALE
- CLINICAL FRAILTY SCALE (CFS)
- INTEGRATED CARE FOR OLDER PEOPLE (ICOPE) SCREENING TOOL

Physical examination
- Vital signs
- Level of consciousness
- Oxygen support
  Pain (VISUAL ANALOGUE SCALE)
- Pressure ulcers

Admission diagnoses
Attending physician prognostic opinion
Recommendations

DISCHARGE

Outcomes
- Length of stay
- Discharge destination
- Death (cause of death)

Complications
- Infections
- Delirium (predominant subtype, precipitating factors, interventions)
- Falls
- Intensive care utilization

Medical summary
- New diagnoses
- Tube feeding
- Surgical procedures
- Discharge medications

Geriatric assessment (items typically reassessed when significantly changed from admission)
- Social support during hospitalization
- ADLs
- 10-CS
- Self-assessed health status
- GDS-4
- Body mass index
- Weight loss history
- Gait speed

© 2026 Avelino-Silva TJ et al. *JAMA Network Open*.

**Patient experience/satisfaction**<br>**Attending physician prognostic opinion**
**FOLLOW-UP**<br>**(phone interviews 30, 90, 180, 270, and 365 days after discharge)**
**Informant identification**<br>**Survival**<br>**Institutionalization**<br><br>**Geriatric assessment**<br>• Living arrangement<br>• Social support<br>• UCLA 3-ITEM LONELINESS SCALE<br>• Use of rehabilitation resources (since the last contact)<br>• History of emergency department visits and hospitalizations (since the last contact)<br>• Falls (since the last contact)<br>• FRAIL Scale<br>• CFS<br>• Reported weight<br>• ADLs<br>• IADLs<br>• 10-CS (180 and 365 days)<br>• Number of medications (365 days)

© 2026 Avelino-Silva TJ et al. *JAMA Network Open*.

eTable 4. Definitions and Assessment Tools for Geriatric Syndromes

Condition	Definition/Assessment Tool
**Loneliness**	UCLA 3-Item Loneliness Scale (UCLA-3) score of 6 or higher (not administered if cognitive impairment precluded completion)[28]
**Dementia**	Clinical Dementia Rating (CDR) score of 1 or higher, based on pre-hospitalization characteristics[25]
**Depressive symptoms**	Assessed using questions from the Integrated Care for Older People (ICOPE) Screening Tool—present if over the past two weeks, feeling down, depressed, or hopeless, or having little interest or pleasure in doing things[31]
**Frailty**	Clinical Frailty Scale (CFS) score of 5 or higher, based on pre-admission characteristics[8]
**Visual and Hearing Impairment**	Assessed using questions from the ICOPE Screening Tool; present if unable to see well enough to recognize a friend across the street, or read in good lighting with glasses, or unable to hear and understand normal speech[31]
**Disability**	Need for assistance in one or more activities of daily living (ADLs) upon admission (bathing, dressing, eating, toileting, and transferring)[29]
**Immobility**	Absent or very limited bed mobility, assessed using the Exton-Smith Scale[26]
**Incontinence**	Urinary and/or fecal incontinence most of the time, assessed using the Exton-Smith Scale[26]
**Falls**	One or more falls in the 12 months prior to hospitalization, assessed using the 10-minute Targeted Geriatric Assessment (10-TaGA)[23]
**Malnutrition**	Score of 7 or less on the Mini-Nutritional Assessment (MNA)[27]
**Pressure Ulcers**	Presence of one or more ulcers of any stage, identified during physical examination
**Polypharmacy**	Regular use of five or more medications, assessed using the 10-TaGA[23]
**Potentially Inappropriate Medications**	Regular use of two or more medications deemed inappropriate by the 2023 American Geriatrics Society Beers Criteria[30]
**Delirium**	Identified using the Confusion Assessment Method (CAM), consolidated at discharge to include both prevalent and incident episodes[24]

© 2026 Avelino-Silva TJ et al. *JAMA Network Open*.

eTable 5. Characteristics of Participating Centers in the CHANGE Study by Country and Region

	Total	Angola	Brazil					Chile	Colombia	Portugal	p-value
	N=43	N=1	*S* N=4	*SE* N=19	*CW* N=2	*NE* N=10	*N* N=3	N=1	N=2	N=1	
**Neighborhood**											0.9
Urban (central region)	37	1	4	16	2	7	3	1	2	1	
Urban (periphery)	6	0	0	3	0	3	0	0	0	0	
**Predominant level of healthcare**											0.55
Secondary	3	0	0	2	0	0	1	0	0	0	
Tertiary	40	1	4	17	2	10	2	1	2	1	
**Predominant healthcare system**											0.61
Public health system	31	0	3	13	2	8	3	1	1	0	
Private healthcare	10	1	1	5	0	1	0	0	1	1	
Equally public and private healthcare	2	0	0	1	0	1	0	0	0	0	
**Teaching hospital**	39	1	3	18	2	8	3	1	2	1	0.7
**Total number of institution beds, median (IQR)**	399 (220, 600)	200 (NA)	277 (232, 573)	447 (287, 600)	355 (311, 399)	271 (238, 750)	80 (80, 102)	645 (NA)	550 (400, 700)	106 (NA)	0.11
**Total number of recruitment unit beds, median (IQR)**	50 (20, 154)	27 (NA)	160 (53, 333)	40 (20, 200)	85 (15, 154)	83 (22, 128)	30 (20, 38)	11 (NA)	55 (50, 60)	100 (NA)	0.74
**Geriatric prevention protocols**											
Delirium prevention	25	0	4	13	1	3	2	1	1	0	0.13
Fall prevention	38	0	4	17	2	8	3	1	2	1	0.48
Pressure ulcers	39	4	18	1	9	2	1	1	2	1	0.37
**Existence of clinical pharmacy service**	38	1	4	16	2	8	3	1	2	1	1
**Type of accommodation**											
Private room (1 patient)	21	1	3	8	1	4	0	1	2	1	0.27
Semi-private room (2 patients)	29	1	3	13	1	5	2	1	2	1	0.96
Ward room (≥3 patients)	27	0	3	8	2	10	2	1	1	0	0.009
**Availability of assistive devices**											
**Cane**											0.7
Yes	12	0	0	7	1	3	0	1	0	0	
No, but recommends bringing from home	22	1	2	9	0	5	2	0	2	1	

© 2026 Avelino-Silva TJ et al. *JAMA Network Open*.

No, nor systematically recommends	9	0	2	3	1	2	1	0	0	0	
**Walker**											0.19
Yes	23	1	1	14	1	3	0	1	1	1	
No, but recommends bringing from home	11	0	2	2	0	4	2	0	1	0	
No, nor systematically recommends	9	0	1	3	1	3	1	0	0	0	
**Glasses**											0.96
Yes	5	0	0	3	0	1	1	0	0	0	
No, but recommends bringing from home	32	1	3	14	2	6	2	1	2	1	
No, nor systematically recommends	6	0	1	2	0	3	0	0	0	0	
**Hearing aids**											0.93
Yes	5	0	0	3	0	1	1	0	0	0	
No, but recommends bringing from home	29	1	3	13	1	5	2	1	2	1	
No, nor systematically recommends	9	0	1	3	1	4	0	0	0	0	
**Dentures**											0.75
Yes	5	0	0	3	0	1	1	0	0	0	
No, but recommends bringing from home	32	1	3	15	1	6	2	1	2	1	
No, nor systematically recommends	6	0	1	1	1	3	0	0	0	0	
**Accessibility**											
Bus	42	1	4	19	2	10	3	1	1	1	0.16
Metro	12	0	0	9	0	2	0	0	0	1	0.22
Free parking	13	1	1	4	2	4	1	0	0	0	0.29
Paid parking	27	0	4	13	0	5	1	1	2	1	0.13
Free ambulance	15	0	1	8	1	3	1		1	0	1.00
Paid ambulance	14	1	1	9	0	1	1	0	0	1	0.18
Ramps and elevators	43	1	4	19	2	10	3	1	2	1	
Adapted bathrooms	42	1	4	19	2	9	3	1	2	1	0.56
Tactile signage	15	0	2	6	1	4	1	0	1	0	0.99
**Existence of nearby hospitals**											0.13
Yes (1 hospital within 5 km)	9	1	1	3	0	1	1	0	2	0	
Yes (>1 hospital within 5 km)	31	0	3	15	2	8	2	0	0	1	
No (no hospitals within 5 km)	3	0	0	1	0	1	0	1	0	0	

N: number of participating centers; S: South; SE: Southeast; CW: Central-West; NE: Northeast; N: North; NA: not applicable

© 2026 Avelino-Silva TJ et al. *JAMA Network Open*.

eTable 6. Baseline Sociodemographic, Health System, and Clinical Case Mix of Hospitalized Older Adults by Country and Region

	Total	Angola	Brazil					Chile	Colombia	Portugal	p-value
			S	*SE*	*CW*	*NE*	*N*				
	(N=2556)	(N=48)	(N=292)	(N=1357)	(N=137)	(N=537)	(N=67)	(N=16)	(N=64	N=38)	
Age, mean year (SD)	79 (9)	78 (7)	76 (8)	80 (9)	85 (6)	78 (8)	76 (10)	75 (7)	88 (6)	80 (8)	<0.001
Sex											0.41
Male	1119 (43.8)	19 (39.6)	128 (43.8)	610 (45.0)	50 (36.5)	227 (42.3)	33 (49.3)	6 (37.5)	25 (39.1)	21 (55.3)	
Female	1437 (56.2)	29 (60.4)	164 (56.2)	747 (55.0)	87 (63.5)	310 (57.7)	34 (50.7)	10 (62.5)	39 (60.9)	17 (44.7)	
Race											<0.001
White	1349 (52.8)	3 (6.2)	225 (77.1)	822 (60.6)	60 (43.8)	192 (35.8)	8 (11.9)	2 (12.5)	0 (0.0)	37 (97.4)	
Black	1139 (44.6)	45 (93.8)	67 (22.9)	488 (36.0)	73 (53.3)	337 (62.8)	58 (86.6)	14 (87.5)	56 (87.5)	1 (2.6)	
Other*	68 (2.7)	0 (0.0)	0 (0.0)	47 (3.5)	4 (2.9)	8 (1.5)	1 (1.5)	0 (0.0)	8 (12.5)	0 (0.0)	
Education, median years (IQR)	5 (3, 9)	2 (0, 4)	5 (4, 8)	5 (4, 10)	2 (0, 4)	5 (1, 10)	5 (2, 7)	6.5 (2.5, 10)	3 (1.5, 5)	12 (5, 15)	<0.001
City's HDI, mean score (SD)	0.789 (0.032)	0.697 (0.000)	0.804 (0.008)	0.805 (0.003)	0.804 (0.010)	0.748 (0.034)	0.743 (0.004)	0.790 (0.000)	0.797 (0.000)	0.835 (0.000)	<0.001
Hospital healthcare system											<0.001
Public	1943 (76.0)	0 (0.0)	268 (91.8)	1051 (77.5)	137 (100.0)	394 (73.4)	67 (100.0)	16 (100.0)	10 (15.6)	0 (0.0)	
Private	505 (19.8)	48 (100.0)	24 (8.2)	287 (21.1)	0 (0.0)	54 (10.1)	0 (0.0)	0 (0.0)	54 (84.4)	38 (100.0)	
Mixed	108 (4.2)	0 (0.0)	0 (0.0)	19 (1.4)	0 (0.0)	89 (16.6)	0 (0.0)	0 (0.0)	0 (0.0)	0 (0.0)	
ED visits or hospitalizations (last 6 months)											<0.001
No	1065 (41.7)	40 (83.3)	129 (44.2)	485 (35.7)	80 (58.4)	249 (46.4)	20 (29.9)	4 (25.0)	38 (59.4)	20 (52.6)	
≥ 1 ED visits (no hospitalization)	649 (25.4)	4 (8.3)	64 (21.9)	367 (27.0)	31 (22.6)	120 (22.3)	26 (38.8)	7 (43.8)	19 (29.7)	11 (28.9)	
≥1 hospitalization	842 (32.9)	4 (8.3)	99 (33.9)	505 (37.2)	26 (19.0)	168 (31.3)	21 (31.3)	5 (31.2)	7 (10.9)	7 (18.4)	
Chronic diseases, median count (IQR)	3 (2, 4)	2 (1, 3)	3 (2, 4)	3 (2, 5)	3 (2, 4)	3 (2, 4)	2 (1, 3)	4 (2, 5)	2 (1.5, 3)	3 (2, 4)	<0.001
NEWS2, median score (IQR)	4 (2, 6)	3 (1, 3)	4 (2, 6)	4 (2, 6)	6 (3, 7)	3 (2, 5)	3 (1, 5)	4 (1, 8)	6 (3.5, 8)	1.5 (0, 3)	<0.001
Geriatric syndromes, median count (IQR)	5 (3, 8)	5 (3, 6.5)	5.5 (3, 7)	6 (3, 8)	7 (5, 8)	5 (3, 7)	3 (2, 5)	4.5 (3, 6)	5 (4, 6)	3 (2, 5)	<0.001

N: number of participants; S: South; SE: Southeast; CW: Central-West; NE: Northeast; N: North; SD: standard deviation; IQR: interquartile range; HDI: Human Development Index; ED: emergency department; NEWS2: National Early Warning Score 2

© 2026 Avelino-Silva TJ et al. *JAMA Network Open*.

eTable 7. Multivariable mixed-effects models relating patient and system characteristics to (A) cumulative geriatric syndrome burden and (B) 90-day all-cause mortality in Brazilian hospitalized older adults (N=2,390).

A. Association between participant characteristics and number of geriatric syndromes				
	**Unadjusted IRR (95%CI)**	**p-value**	**Adjusted IRR (95%CI)**	**p-value**
Age (per 5 years)	1.086 (1.073, 1.100)	<0.001	1.085 (1.072, 1.098)	<0.001
Sex				
Male	Referent		Referent	
Female	1.111 (1.066, 1.157)	<0.001	1.072 (1.032, 1.114)	<0.001
Race				
Not Black	Referent		Referent	
Black	0.972 (0.928, 1.018)	0.23	0.989 (0.947, 1.032)	0.61
Education (per 4 years)	0.950 (0.931, 0.968)	<0.001	0.976 (0.958, 0.994)	0.010
HDI (per 0.1 unit)	1.441 (1.146, 1.811)	0.002	1.295 (1.093, 1.533)	0.003
Healthcare system				
Private or mixed	Referent		Referent	
Public	1.062 (0.895, 1.260)	0.49	1.133 (1.004, 1.278)	0.043
ED visits or hospitalizations (last 6 months)				
No	Referent		Referent	
≥ 1 ED visits (no hospitalization)	1.214 (1.154, 1.277)	<0.001	1.189 (1.134, 1.248)	<0.001
≥1 hospitalization	1.342 (1.281, 1.406)	<0.001	1.333 (1.274, 1.393)	<0.001
Number of chronic diseases*	1.041 (1.030, 1.051)	<0.001	1.028 (1.018, 1.038)	<0.001

B. Association between number of geriatric syndromes and 90-day mortality†				
	**Unadjusted HR (95%CI)**	**p-value**	**Adjusted HR (95%CI)**	**p-value**
Geriatric syndromes (count)	1.230 (1.193, 1.269)	<0.001	1.198 (1.130, 1.269)	<0.001
Age (5 years)	1.171 (1.112, 1.233)	<0.001	1.091 (1.034, 1.152)	0.002
Sex				
Male	Referent		Referent	
Female	0.846 (0.716, 0.999)	0.049	0.741 (0.624, 0.879)	<0.001
Race				
Not Black	Referent		Referent	
Black	0.932 (0.778, 1.117)	0.45	0.972 (0.807, 1.170)	0.76
Education (4 years)	0.936 (0.864, 1.014)	0.11	1.060 (0.978, 1.150)	0.16
HDI (per 0.1 unit)	1.394 (0.738, 2.633)	0.31	1.048 (0.650, 1.692)	0.85
Healthcare system				
Private or mixed	Referent		Referent	
Public health system	3.050 (2.102, 4.426)	<0.001	4.175 (2.615, 6.664)	<0.001
ED visits or hospitalizations (last 6 months)				
No	Referent		Referent	
≥ 1 ED visits (no hospitalization)	1.237 (0.995, 1.537)	0.06	1.008 (0.807, 1.260)	0.94
≥1 hospitalization	1.489 (1.219, 1.819)	<0.001	1.192 (0.968, 1.469)	0.10
Number of chronic diseases*	0.956 (0.914, 0.999)	0.047	0.915 (0.875, 0.956)	<0.001
NEWS2 Score	1.175 (1.144, 1.207)	<0.001	1.116 (1.082, 1.151)	<0.001

N: number of participants; IRR: incidence-rate ratios; CI: confidence interval; HR: hazard ratio; HDI: Human Development Index; NEWS2: National Early Warning Score 2

Analyses were performed using: (A) mixed-effects negative binomial regression models with random intercepts at the state/province and hospital levels; and (B) Cox proportional hazards models with random intercepts at the state/province and hospital levels, and a random hospital-level slope for geriatric syndrome count. Incidence-rate ratios (IRRs) represent the change in the outcome (i.e., number of geriatric syndromes), while hazard ratios (HRs) represent the risk of the outcome (i.e., 90-day mortality). The multivariable model adjusts for all listed covariates.

*Number of chronic diseases among stroke, anxiety, asthma, transient ischemic attack, depression, diabetes, chronic kidney disease, connective tissue disease, chronic obstructive pulmonary disease, atrial fibrillation, liver disease, hypertension, myocardial infarction, vascular disease, heart failure, coronary artery disease, hematologic cancer, other cancers, osteoarthritis, osteoporosis, HIV, and peptic ulcer disease

† Coefficients for covariates other than the geriatric-syndrome count are shown to characterize conditional risk gradients and to document the adjustment set; because they are estimated from models that condition on other factors, they should not be interpreted as estimates of the total causal effects of those predictors.

© 2026 Avelino-Silva TJ et al. *JAMA Network Open*.

eTable 8. Modification of the Association of Geriatric Syndrome Burden With 90-Day Mortality by Age

A. Age-specific hazard ratio per additional geriatric syndrome (N=2,556)			
**Age group (years)**	**Adj. HR**	**95%CI**	**p-value**
65–74 (Referent)	1.15	1.07–1.23	<0.001
75–84	1.24	1.15–1.33	<0.001
≥85	1.30	1.20–1.41	<0.001

B. Comparison of high vs. low geriatric syndrome burden across age strata (N=1,414)

Age group (years)	<5 Geriatric syndromes		>8 Geriatric syndromes		Adjusted HR (95%CI) for high geriatric burden within age strata
	N dead/ alive	Adj. HR (95%CI)	N dead/ alive	Adj. HR (95%CI)	
65–74 (Referent)	59/410	1.0	25/57	2.30 (1.30–4.07)	2.30 (1.30–4.07)
75–84	31/321	0.87 (0.56–1.36)	56/93	3.33 (2.02–5.51)	3.83 (2.23–6.57)
≥85	17/156	1.07 (0.60–1.89)	91/98	5.45 (3.36–8.84)	5.10 (2.81–9.26)

Adj.: adjusted; HR: hazard ratio; CI: confidence interval
(A) Adjusted HR are from a mixed-effects Cox proportional hazards model including a product term between continuous syndrome count and three-level age category (65–74, 75–84, ≥85 years), with random intercepts for state and hospital and a hospital-level random slope for syndrome count. Models are adjusted for sex, race (Black vs. other), years of education, municipal Human Development Index, public vs. private health system, prior hospital or emergency department use, and NEWS2 score at admission. In the reference age group (65–74 years), the adjusted HR per additional syndrome was 1.15 (95%CI=1.07–1.23). The corresponding interaction terms for ages 75–84 and ≥85 years were HR multipliers of 1.08 (95%CI=1.00–1.16; p=0.042) and 1.14 (95%CI=1.04–1.24; p=0.004).
(B) Analysis restricted to patients with <5 or >8 geriatric syndromes (N=1,414). Adjusted hazard ratios are from a mixed-effects Cox proportional hazards model including a product term between high vs. low syndrome burden and age category (65–74, 75–84, ≥85 years), with the same covariate and random-effects structure as above (A). Measure of effect modification on additive scale: the relative excess risk due to interaction (RERI) for the joint presence of age ≥85 years and high syndrome burden was 3.09 (95%CI=1.05–5.12; p=0.003). Measure of effect modification on multiplicative scale (expressed as the ratio of HRs comparing high vs. low burden in the oldest vs. youngest age group): HR=2.22 (95%CI=1.07–4.62, p=0.032). These measures indicate positive effect modification by age on both the additive and multiplicative scales.

© 2026 Avelino-Silva TJ et al. *JAMA Network Open*.
