# Supplementary material for: Geriatric Syndromes and Mortality Among Hospitalized Older Adults
Source: JAMA Netw Open. 2026 Jan 27;9(1):e2555740. doi: 10.1001/jamanetworkopen.2025.55740 (PMC12848630; doi:10.1001/jamanetworkopen.2025.55740)
Supplement: Supplement 2. — Nonauthor Collaborators [file jamanetwopen-e2555740-s002.pdf]

Supplemental Online Content: Nonauthor Collaborators
*First name, last name, and suffix (if applicable) are required and will appear in PubMed.

| *Group Name(s): CHANGE Study Group | | | | | | | |
|---|---|---|---|---|---|---|---|
| ***First Name and Middle Initial(s)** | ***Last Name** | ***Suffix (eg, Jr, III)** | Academic Degrees | Institution | Location (city, state/province, country) | Role or Contribution, eg, chair, principal investigator | Group (if more than 1 Group listed in the byline) and/or Subgroup (eg, Steering Committee) |
| Ivan M. | de Oliveira | | MD | Beneficência Portuguesa de São Paulo | São Paulo, SP, Brazil | | |
| Diana | Francisco | | MD | Clínica Girassol | Luanda, Angola | | |
| Eunice | Andrade | | MD | Clínica Girassol | Luanda, Angola | | |
| Nazareth | Neto | | MD | Clínica Girassol | Luanda, Angola | | |
| Nidia | Van Dunem | | MD | Clínica Girassol | Luanda, Angola | | |
| Beatriz N. | da Cunha | | MD | Hospital Central da Irmandade da Santa Casa de Misericórdia de São Pa | São Paulo, SP, Brazil | | |
| Ewerton | Miyadahira | | MD | Hospital Central da Irmandade da Santa Casa de Misericórdia de São Pa | São Paulo, SP, Brazil | | |
| Gustavo M. | Mil Homens | | MD | Hospital Central da Irmandade da Santa Casa de Misericórdia de São Pa | São Paulo, SP, Brazil | | |
| Lisa L. | Mello | | MD | Hospital Central da Irmandade da Santa Casa de Misericórdia de São Pa | São Paulo, SP, Brazil | | |
| Mariana M. | Teruya | | MD | Hospital Central da Irmandade da Santa Casa de Misericórdia de São Pa | São Paulo, SP, Brazil | | |
| Mario S. S. | Cabral | | MD | Hospital Central da Irmandade da Santa Casa de Misericórdia de São Pa | São Paulo, SP, Brazil | | |
| Matheus P. | Viola | | MD | Hospital Central da Irmandade da Santa Casa de Misericórdia de São Pa | São Paulo, SP, Brazil | | |
| Renato T. | Galvão | | MD | Hospital Central da Irmandade da Santa Casa de Misericórdia de São Pa | São Paulo, SP, Brazil | | |
| Beatriz N. | Nassif | | MD | Hospital das Clínicas da Faculdade de Medicina da USP | São Paulo, SP, Brazil | | |
| Dannielle M. | Guimarães | | MD | Hospital das Clínicas da Faculdade de Medicina da USP | São Paulo, SP, Brazil | | |
| Ezemir D. | Fernandes | Junior | MD | Hospital das Clínicas da Faculdade de Medicina da USP | São Paulo, SP, Brazil | | |
| Flavia A. | de Amorim | | MD | Hospital das Clínicas da Faculdade de Medicina da USP | São Paulo, SP, Brazil | | |
| Flavia | Campora | | MD | Hospital das Clínicas da Faculdade de Medicina da USP | São Paulo, SP, Brazil | | |
| Flavia T. T. | Nakamura | | MD | Hospital das Clínicas da Faculdade de Medicina da USP | São Paulo, SP, Brazil | | |
| Jose S. | Cardoso | Neto | MD | Hospital das Clínicas da Faculdade de Medicina da USP | São Paulo, SP, Brazil | | |
| Julia M. | Menezes | | MD | Hospital das Clínicas da Faculdade de Medicina da USP | São Paulo, SP, Brazil | | |
| Luis E. M. | Martins | | MD | Hospital das Clínicas da Faculdade de Medicina da USP | São Paulo, SP, Brazil | | |
| Mayara V. | Batista | | MD | Hospital das Clínicas da Faculdade de Medicina da USP | São Paulo, SP, Brazil | | |
| Raiza T. | Lira | | MD | Hospital das Clínicas da Faculdade de Medicina da USP | São Paulo, SP, Brazil | | |
| Silvio C. | Amorim | | MD | Hospital das Clínicas da Faculdade de Medicina da USP | São Paulo, SP, Brazil | | |
| Vitor A. | Fontenelles | | MD | Hospital das Clínicas da Faculdade de Medicina da USP | São Paulo, SP, Brazil | | |
| Gabriela M. | Costa | | - | Hospital das Clínicas da Faculdade de Medicina de Botucatu | Botucatu, SP, Brazil | | |
| Julio C. | Moriguti | | MD, PhD | Hospital das Clínicas da Faculdade de Medicina de Ribeirão Preto | Ribeirão Preto, SP, Brazil | | |
| Edgar N. | de Moraes | | MD, PhD | Hospital das Clínicas da Universidade Federal de Minas Gerais | Belo Horizonte, MG, Brazil | | |
| Tatiana C. E. | Pinheiro | | MD, MS | Hospital das Clínicas da Universidade Federal de Minas Gerais | Belo Horizonte, MG, Brazil | | |
| Alicia R. M. | Accioly | | MD, MSc | Hospital das Clínicas da Universidade Federal de Pernambuco | Recife, PE, Brazil | | |
| Hugo O. D. M. | Gomes | | MD | Hospital das Clínicas da Universidade Federal de Pernambuco | Recife, PE, Brazil | | |
| Mariana A. | de Luna | | MD | Hospital das Clínicas da Universidade Federal de Pernambuco | Recife, PE, Brazil | | |
| Mayara S. | Honorato | | MD | Hospital das Clínicas da Universidade Federal de Pernambuco | Recife, PE, Brazil | | |
| Milena B. A. | Silva | | MD | Hospital das Clínicas da Universidade Federal de Pernambuco | Recife, PE, Brazil | | |
| Rebeca M. S. | Coelho | | MD | Hospital das Clínicas da Universidade Federal de Pernambuco | Recife, PE, Brazil | | |
| Rosana S. | Batista | | MD | Hospital das Clínicas da Universidade Federal de Pernambuco | Recife, PE, Brazil | | |
| Andre | Fattori | | MD, PhD | Hospital de Clínicas - UNICAMP | Campinas, SP, Brazil | | |
| Estela F. | Vilela | | MD | Hospital de Clínicas - UNICAMP | Campinas, SP, Brazil | | |

Supplemental Online Content: Nonauthor Collaborators
*First name, last name, and suffix (if applicable) are required and will appear in PubMed.

| ***First Name and Middle Initial(s)** | ***Last Name** | ***Suffix (eg, Jr, III)** | Academic Degrees | Institution | Location (city, state/province, country) | Role or Contribution, eg, chair, principal investigator | Group (if more than 1 Group listed in the byline) and/or Subgroup (eg, Steering Committee) |
|---|---|---|---|---|---|---|---|
| Jessica | Valonini | | MD | Hospital de Clínicas - UNICAMP | Campinas, SP, Brazil | | |
| Rodolfo A. O. | Nogueira | | MD | Hospital de Clínicas - UNICAMP | Campinas, SP, Brazil | | |
| Emilio H. | Moriguchi | | MD, MSc,PhD | Hospital de Clínicas de Porto Alegre | Porto Alegre, RS, Brazil | | |
| Francine F. | Klein | | MSc, PhD | Hospital de Clínicas de Porto Alegre | Porto Alegre, RS, Brazil | | |
| Aline T. S. | Santos | | MD | Hospital do Coração de Natal | Natal, RN, Brazil | | |
| Giovanni G. N. | Santos | | MD | Hospital do Coração de Natal | Natal, RN, Brazil | | |
| João G. A. | de Lima | | - | Hospital do Coração de Natal | Natal, RN, Brazil | | |
| Juliana C. | de Souza | | MD | Hospital do Coração de Natal | Natal, RN, Brazil | | |
| Juliano S. | de Araujo | | MD | Hospital do Coração de Natal | Natal, RN, Brazil | | |
| Maria C. T. | Vianna | | - | Hospital do Coração de Natal | Natal, RN, Brazil | | |
| Natalia C. | Guedes | | MD | Hospital do Coração de Natal | Natal, RN, Brazil | | |
| Rafael V. S. | Barreto | | - | Hospital do Coração de Natal | Natal, RN, Brazil | | |
| Raphael A. | Filgueiras | | - | Hospital do Coração de Natal | Natal, RN, Brazil | | |
| Rayane L. C. D. | de Medeiros | | - | Hospital do Coração de Natal | Natal, RN, Brazil | | |
| Brunna S. | Oliveira | | PharmD | Hospital Geral de Vitória da Conquista | Vitória da Conquista, BA, Brazil | | |
| Esther S. M. | Melo | | Phychologist | Hospital Geral de Vitória da Conquista | Vitória da Conquista, BA, Brazil | | |
| Hellen M. M. | Cardoso | | Phychologist | Hospital Geral de Vitória da Conquista | Vitória da Conquista, BA, Brazil | | |
| Iuri C. | Gusmão | | - | Hospital Geral de Vitória da Conquista | Vitória da Conquista, BA, Brazil | | |
| Jonatas S. P. | Porto | | MD | Hospital Geral de Vitória da Conquista | Vitória da Conquista, BA, Brazil | | |
| Marcio G. G. | de Oliveira | | PharmD, MSc, PhD | Hospital Geral de Vitória da Conquista | Vitória da Conquista, BA, Brazil | | |
| Maria E. S. G. | Roberto | | MD | Hospital Geral de Vitória da Conquista | Vitória da Conquista, BA, Brazil | | |
| Nara L. F. | Rebouças | | - | Hospital Geral de Vitória da Conquista | Vitória da Conquista, BA, Brazil | | |
| Roberta B. | Jauris | | Psychologist, MSc | Hospital Geral de Vitória da Conquista | Vitória da Conquista, BA, Brazil | | |
| Tatiane D. C. | Valença | | PT, MSc, PhD | Hospital Geral de Vitória da Conquista | Vitória da Conquista, BA, Brazil | | |
| Andrezza M. | Fernandes | | MD | Hospital Geral Dr. César Cals de Oliveira | Fortaleza, CE, Brazil | | |
| Carolina M. | Feijo | | MD | Hospital Geral Dr. César Cals de Oliveira | Fortaleza, CE, Brazil | | |
| Hellen M. P. | Rocha | | RN | Hospital Geral Dr. César Cals de Oliveira | Fortaleza, CE, Brazil | | |
| Lara A. | Vieira | | MD | Hospital Geral Dr. César Cals de Oliveira | Fortaleza, CE, Brazil | | |
| Lirenna P. | Narciso | | MD | Hospital Geral Dr. César Cals de Oliveira | Fortaleza, CE, Brazil | | |
| Luisa B. | Bruno | | MD | Hospital Geral Dr. César Cals de Oliveira | Fortaleza, CE, Brazil | | |
| Nadedja L. Q. | Rocha | | MD | Hospital Geral Dr. César Cals de Oliveira | Fortaleza, CE, Brazil | | |
| Priscila P. S. | Nogueira | | MD | Hospital Geral Dr. César Cals de Oliveira | Fortaleza, CE, Brazil | | |
| Rafael S. B. | Pinheiro | | MD | Hospital Geral Dr. César Cals de Oliveira | Fortaleza, CE, Brazil | | |
| Wallena C. | Brito | | MD | Hospital Geral Dr. César Cals de Oliveira | Fortaleza, CE, Brazil | | |
| Adriana | Alves | | RN | Hospital Israelita Albert Einstein | São Paulo, SP, Brazil | | |
| Julia M. | Menezes | | MD | Hospital Israelita Albert Einstein | São Paulo, SP, Brazil | | |
| Margarete C. P. | Miralia | | RN | Hospital Israelita Albert Einstein | São Paulo, SP, Brazil | | |
| Victor J. D. | Melo | | MD | Hospital Israelita Albert Einstein | São Paulo, SP, Brazil | | |
| Vanessa A. L. | Pires | | RN | Hospital Israelita Albert Einstein | São Paulo, SP, Brazil | | |
| Filipe | Basto | | MD | Hospital Lusíadas Porto | Porto, Portugal | | |

*First name, last name, and suffix (if applicable) are required and will appear in PubMed.

| ***First Name and Middle Initial(s)** | ***Last Name** | ***Suffix (eg, Jr, III)** | Academic Degrees | Institution | Location (city, state/province, country) | Role or Contribution, eg, chair, principal investigator | Group (if more than 1 Group listed in the byline) and/or Subgroup (eg, Steering Committee) |
|---|---|---|---|---|---|---|---|
| Manuela O. C. | Magalhães | | MS, PhD | Hospital Santo Antônio - Obras Sociais Irmã Dulce | Salvador, BA, Brazil | | |
| Dominique K. B. | Silva | | MD | Hospital Santo Antônio - Obras Sociais Irmã Dulce | Salvador, BA, Brazil | | |
| Paula L. | Ferreia | | MD | Hospital Santo Antônio - Obras Sociais Irmã Dulce | Salvador, BA, Brazil | | |
| Rísia M. O. | Barreto | | MD | Hospital Santo Antônio - Obras Sociais Irmã Dulce | Salvador, BA, Brazil | | |
| Camila F. | Lima | | MD | Hospital Santo Antônio - Obras Sociais Irmã Dulce | Salvador, BA, Brazil | | |
| Tatiana S. | Moreira | | MD | Hospital Santo Antônio - Obras Sociais Irmã Dulce | Salvador, BA, Brazil | | |
| Josecy M. S. | Peixoto | | MS, PhD | Hospital Santo Antônio - Obras Sociais Irmã Dulce | Salvador, BA, Brazil | | |
| Alayne M. T. D. | Yamada | | PhD | Hospital São Camilo | São Paulo, SP, Brazil | | |
| Fabio A. | Bittencourt | | MD | Hospital São Camilo | São Paulo, SP, Brazil | | |
| Graziela B. B. | Ivanov | | MD | Hospital São Camilo | São Paulo, SP, Brazil | | |
| Mara G. M. | Silveira | | MD | Hospital São Camilo | São Paulo, SP, Brazil | | |
| Debora D. | Casagrande | | MD | Hospital São José | Criciúma, SC, Brazil | | |
| Thatiana | Dal Toe | | MD,MS | Hospital São José | Criciúma, SC, Brazil | | |
| Eduardo C. | Cruz | | MD | Hospital São Paulo - UNIFESP | São Paulo, SP, Brazil | | |
| Bruna M. | de Carvalho | | MD | Hospital Sírio Libanês | São Paulo, SP, Brazil | | |
| Michel S. | Dantas | | RPh | Hospital Sírio Libanês | São Paulo, SP, Brazil | | |
| Naira H. S. L. | Hojaij | | MD, PhD | Hospital Sírio Libanês | São Paulo, SP, Brazil | | |
| Julia F. | Brenny | | - | Hospital Universitário da Universidade Estadual de Londrina | Londrina, PR, Brazil | | |
| Pedro H. A. | Silva | | - | Hospital Universitário da Universidade Estadual de Londrina | Londrina, PR, Brazil | | |
| Erika C. N. | Giuliano | | - | Hospital Universitário da Universidade Federal de São Carlos | São Carlos, SP, Brazil | | |
| Amarildo B. S. | Oliveira | | MD | Hospital Universitário de Brasília | Brasília, DF, Brazil | | |
| Antonio L. | Sarmento | Filho | MD | Hospital Universitário de Brasília | Brasília, DF, Brazil | | |
| Einstein F. | de Camargos | | MD, MSc, PhD | Hospital Universitário de Brasília | Brasília, DF, Brazil | | |
| Larissa F. L. | e Abreu | | MD | Hospital Universitário de Brasília | Brasília, DF, Brazil | | |
| Luciana L. L. | Martini | | MD, MSc, PhD | Hospital Universitário de Brasília | Brasília, DF, Brazil | | |
| Vanessa S. | Canossa | | MD | Hospital Universitário de Brasília | Brasília, DF, Brazil | | |
| Yan B. | Jardim | | - | Hospital Universitário de Brasília | Brasília, DF, Brazil | | |
| Karlo E. | Moreira | | - | Hospital Universitário João de Barros Barreto | Belém, PA, Brazil | | |
| Eliana | Pineda | | MD, MSc | Hospital Universitario Mayor Méderi | Bogotá, Colombia | | |
| Elly | Morros | | MD | Hospital Universitario Mayor Méderi | Bogotá, Colombia | | |
| Catarina R. F. | do Nascimento | | MD | Hospital Universitário Onofre Lopes | Natal, RN, Brazil | | |
| Marconi E. | Maia | Junior | MD | Hospital Universitário Onofre Lopes | Natal, RN, Brazil | | |
| Carla M. | Ribeiro | | MD | Hospital Universitário Pedro Ernesto | Rio de Janeiro, RJ, Brazil | | |
| Marilia G. S. | Torre | | MD | Hospital Universitário Pedro Ernesto | Rio de Janeiro, RJ, Brazil | | |
| Nathalia | Gomes | | MD | Hospital Universitário Pedro Ernesto | Rio de Janeiro, RJ, Brazil | | |
| Christiane M. | Santana | | MD | Hospital Universitário Professor Edgard Santos | Salvador, BA, Brazil | | |
| Fabia S. O. | Junqueira | | - | Hospital Universitário Professor Edgard Santos | Salvador, BA, Brazil | | |
| Manuela O. C. | Magalhães | | MD, PhD | Hospital Universitário Professor Edgard Santos | Salvador, BA, Brazil | | |
| Murilo S. S. | Passos | | - | Hospital Universitário Professor Edgard Santos | Salvador, BA, Brazil | | |
| Ronald C. | Gomez | | MD, MSc | Hospital Universitario San Ignacio | Bogotá, Colombia | | |

Supplemental Online Content: Nonauthor Collaborators
*First name, last name, and suffix (if applicable) are required and will appear in PubMed.

| ***First Name and Middle Initial(s)** | ***Last Name** | ***Suffix (eg, Jr, III)** | Academic Degrees | Institution | Location (city, state/province, country) | Role or Contribution, eg, chair, principal investigator | Group (if more than 1 Group listed in the byline) and/or Subgroup (eg, Steering Committee) |
|---|---|---|---|---|---|---|---|
| Ana L. | Kanaji | | MD, PhD | Instituto do Câncer do Estado de São Paulo | São Paulo, SP, Brazil | | |
| Andreyna J. | Rodrigues | | RN, MSN | Real Hospital Português de Beneficência | Recife, PE, Brazil | | |
| Camila M. F. D. | Ferreira | | MD | Real Hospital Português de Beneficência | Recife, PE, Brazil | | |
| Iolanda G. R. | de Oliveira | | MD | Real Hospital Português de Beneficência | Recife, PE, Brazil | | |
| Ivo B. S. | Silva | | MD | Real Hospital Português de Beneficência | Recife, PE, Brazil | | |
| Luciulo | Melo | | MD, MSc | Real Hospital Português de Beneficência | Recife, PE, Brazil | | |
| Milena M. | dos Santos | | RN | Real Hospital Português de Beneficência | Recife, PE, Brazil | | |
| Walter A. | de Araujo | Junior | MD | Real Hospital Português de Beneficência | Recife, PE, Brazil | | |
| Vanessa F. R. | Saraiva | | MD | Real Hospital Português de Beneficência | Recife, PE, Brazil | | |
| Flavio F. | Arbex | | MD, PhD | Santa Casa de Araraquara | Araraquara, SP, Brazil | | |
| Maria J. C. | Souza | | MD, MS | Santa Casa de Araraquara | Araraquara, SP, Brazil | | |
| Laiane M. | Dias | | MD,PhD | Hospital Jean Bitar | Belém,PA, Brazil | | |
