# Supplementary material for: Geriatric Syndromes and Mortality Among Hospitalized Older Adults
Source: JAMA Netw Open. 2026 Jan 27;9(1):e2555740. doi: 10.1001/jamanetworkopen.2025.55740 (PMC12848630; doi:10.1001/jamanetworkopen.2025.55740)
Supplement: Supplement 3. — Data Sharing Statement [file jamanetwopen-e2555740-s003.pdf]

Data Sharing Statement

Data

Data available: Deidentified individual participant data (including a data dictionary and analysis code) will be made available upon reasonable request to the corresponding author, beginning 12 months after publication, for researchers with methodologically sound proposals and a signed data use agreement. Requests will be reviewed by the CHANGE Steering Committee.
